# Albert Renold Memorial Lecture: Molecular Background of Nutritionally Induced Insulin Resistance Leading to Type 2 Diabetes – From Animal Models to Humans

**DOI:** 10.1155/EDR.2001.299

**Published:** 2001

**Authors:** Eleazar Shafrir

**Affiliations:** 1 Department of Biochemistry Hadassah University Hospital and Hebrew University-Hadassah Medical School Jerusalem Israel

## Abstract

Albert Renold strived to gain insight into the abnormalities of human diabetes by defining the pathophysiology of the disease peculiar to a given animal. He investigated the Israeli desert-derived spiny mice (Acomys cahirinus), which became obese on fat-rich seed diet. After a few months hyperplasia and hypertrophy of *β*-cells occurred leading to a sudden rupture, insulin loss and ketosis. Spiny mice were low insulin responders, which is probably a characteristic of certain desert animals, protecting against insulin oversecretion when placed on an abundant diet. We have compared the response to overstimulation of several mutant diabetic species and nutritionally induced nonmutant animals when placed on affluent diet. Some endowed with resilient *β*-cells sustain long-lasting oversecretion, compensating for the insulin resistance, without lapsing into overt diabetes. Some with labile beta cells exhibit apoptosis and lose their capacity of coping with insulin resistance after a relatively short period. The wide spectrum of response to insulin resistance among different diabetes prone species seems to represent the varying response of human beta cells among the populations. In search for the molecular background of insulin resistance resulting from overnutrition we have studied the Israeli desert gerbil Psammomys obesus (sand rat), which progresses through hyperinsulinemia, followed by hyperglycemia and irreversible beta cell loss. Insulin resistance was found to be the outcome of reduced activation of muscle insulin receptor tyrosine kinase by insulin, in association with diminished GLUT4 protein and DNA content and overexpression of PKC isoenzymes, notably of PKC*ε*. This overexpression and translocation to the membrane was discernible even prior to hyperinsulinemia and may reflect the propensity to diabetes in nondiabetic species and represent a marker for preventive action. By promoting the phosphorylation of serine/threonine residues on certain proteins of the insulin signaling pathway, PKC*ε* exerts a negative feedback on insulin action. PKC*ε* was also found to attenuate the activity of PKB and to promote the degradation of insulin receptor, as determined by co-incubation in HEK 293 cells. PKC*ε* overexpression was related to the rise in muscle diacylglycerol and lipid content, which are prevalent on lascivious nutrition especially if fat-rich. Thus, Psammomys illustrates the probable antecedents of the development of worldwide diabetes epidemic in human populations emerging from food scarcity to nutritional affluence, inappriopriate to their metabolic capacity.


*Int. Jnl. Experimental Diab. Res.*, Vol. 2, pp. 299–319
Reprints available directly from the publisher
Photocopying permitted by license only

© 2001 OPA (Overseas Publishers Association) N.V.
Published by license under
the Harwood Academic Publishers imprint,
part of Gordon and Breach Publishing,
a member of the Taylor & Francis Group.
Printed in the U.S.A.

### Albert Renold Memorial Lecture: Molecular Background of Nutritionally Induced Insulin Resistance Leading to Type 2 Diabetes – From Animal Models to Humans

ELEAZAR SHAFRIR

*Department of Biochemistry, Hadassah University Hospital and Hebrew University-Hadassah Medical School, Jerusalem, Israel*

Presented at the 8th International Workshop on Lessons from Animal Diabetes, Tokyo, 24–26 July, 2001

**Albert Renold strived to gain insight into the abnormalities of human diabetes by defining the pathophysiology of the disease peculiar to a given animal. He investigated the Israeli desert-derived spiny mice (Acomys cahirinus), which became obese on fat-rich seed diet. After a few months hyperplasia and hypertrophy of $\beta$-cells occurred leading to a sudden rupture, insulin loss and ketosis. Spiny mice were low insulin responders, which is probably a characteristic of certain desert animals, protecting against insulin oversecretion when placed on an abundant diet. We have compared the response to overstimulation of several mutant diabetic species and nutritionally induced nonmutant animals when placed on affluent diet. Some endowed with resilient $\beta$-cells sustain long-lasting oversecretion, compensating for the insulin resistance, without lapsing into overt diabetes. Some with labile beta cells exhibit apoptosis and lose their capacity of coping with insulin resistance after a relatively short period. The wide spectrum of response to insulin resistance among different diabetes prone species seems to represent the varying response of human beta cells among the populations. In search for the molecular background of insulin resistance resulting from overnutrition we have studied the Israeli desert gerbil Psammomys obesus (sand rat), which progresses through hyperinsulinemia, followed by hyperglycemia and irreversible beta cell loss. Insulin resistance was found to be the outcome of reduced activation of muscle insulin receptor tyrosine kinase by insulin, in association with diminished GLUT4 protein and DNA content and overexpression of PKC isoenzymes, notably of PKC$\varepsilon$. This overexpression and translocation to the membrane was discernible even prior to hyperinsulinemia and may reflect the propensity to diabetes in nondiabetic species and represent a marker for preventive action. By promoting the phosphorylation of serine/threonine residues on certain proteins of the insulin signaling pathway, PKC$\varepsilon$ exerts a negative feedback on insulin action. PKC$\varepsilon$ was also found to attenuate the activity of PKB and to promote the degradation of insulin receptor, as determined by co-incubation in HEK 293 cells. PKC$\varepsilon$ overexpression was related to the rise in muscle diacylglycerol and lipid content, which are prevalent on lascivious nutrition especially if fat-rich. Thus, Psammomys illustrates the probable antecedents of the development of worldwide diabetes epidemic in human populations emerging from food scarcity to nutritional affluence, inappriopriate to their metabolic capacity.**

*Keywords*: Insulin resistance; Hyperinsulinemia; Insulin receptor; Tyrosine kinase; GLUT4 transporter; Protein kinase C; Protein kinase B; Diacylglycerol phosphate; *Psammomys obesus* (sand rat); *Acomys cahirinus* (Spiny mouse)

*Abbreviations*: DAG, diacyl glycerol; DP, diabetes prone; DR, diabetes resistant; FFA, free fatty acids; HE, high energy; HEK, human embryonic kidney cells; IGT, impaired glucose tolerance; IR, insulin receptor; IRS, insulin receptor substrate; LE, low energy; P13K, phosphoinositol-3-kinase; PEPCK, phosphoenolpyruvate carboxykinase; PKB, protein kinase B; PKC, protein kinase C; PTPase, protein tyrosine phosphatase; SDS-PAGE, sodium dodecyl sulfate polyacrylamide gel electrophoresis; TG, triglycerides; TK, tyrosine kinase; TPA, tetradecanoyl phorbol acetate

### INTRODUCTION

This lecture is dedicated to the memory of Albert Renold (Fig. 1). I shall not dwell on his biography, which is well known to most of us. I would like just to say that he was endowed with a generous personality, free of any scientific or personal prejudice, and unbound enthusiasm for experimental research of diabetes. His leadership capacity, art of dialogue and negotiations, enabled him to establish the EASD in 1965 and perform outstandingly as the President of IDF 1979–1983. He attracted a cohort of renowned international scientists to his Geneva Department, and created there a European Mecca of diabetes research and teaching from which close to 500 outstanding contributions emanated. He passed away suddenly on March 21, 1988, about 13 years ago.

I had the privilege to share with him the excitement of experimental diabetes research, in animal models and to launch in 1982 the International Workshops on Lessons from Animal Diabetes in Jerusalem, (Fig. 2) which have continued to take place in several locations, and today, the 8th Workshop, in Tokyo.

Albert Renold strived to gain insight into the abnormalities of human diabetes by defining the pathophysiology of the disease peculiar to a given animal. He was convinced that the elucidation of pathogenesis of human diabetes will be better understood by the integration of pathogenesis of several genetically transmitted or experimental diabetes syndromes. His belief was that whether induced by surgical, chemical, endocrine, immunologic or genetic treatments, models of diabetes can be extremely informative and helpful, but may be misinterpreted by equating a single model with human diabetes.[1] With the advances in research, it may be said that the existence of numerous variants of animals with characteristics close to Type 2 diabetes allows to uncover different mechanisms leading to insulin resistance, β-cell demise, disrupted glucoregulation and species specific complications. Consolidation of this knowledge may pave the way to classification of human diabetes into better defined entities.

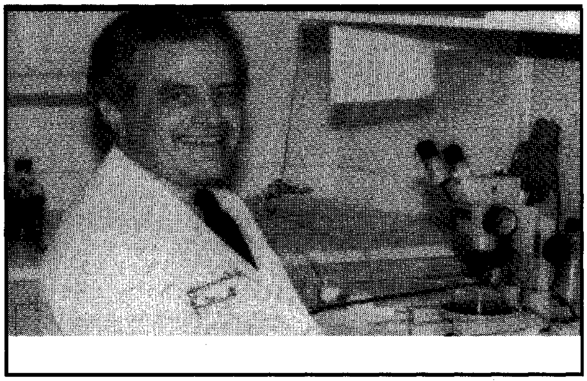

FIGURE 1 Professor Albert E. Renold in his laboratory at the Institute de Biochimie Clinique in Geneva.

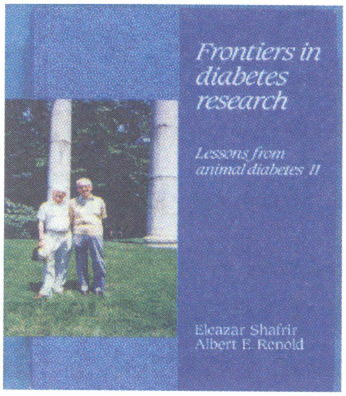

FIGURE 2 Professors A. E. Renold and E. Shafrir with Vol. 1 of the Lessons from Animal Diabetes book series in the background.

Among the models Renold investigated were the Israeli desert-derived spiny mice (*Acomys cahirinus*), (Fig. 3) which became obese on fat-rich seed diet. After a few months β-cell hyperplasia and hypertrophy developed leading to a sudden islet rupture and resulting in ketosis and insulin deficiency. Prior to overt diabetes spiny mice exhibited only intermittent hyperglycemia and impaired glucose tolerance. They were characterized as "low insulin responders". Different secretagogues failed to elicit sufficient β-cell insulin response. This was attributed to several anatomic and biochemical features of their β-cells, *e.g.*, low adenylate cyclase activity, low

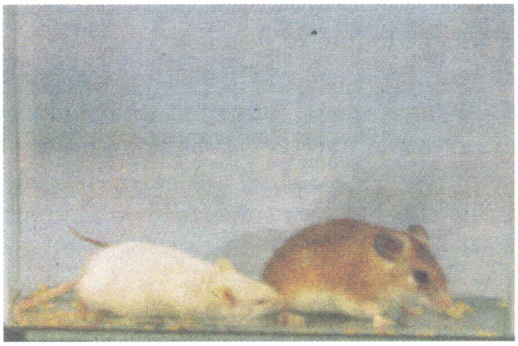

FIGURE 3 A spiny mouse (*Acomys cahirinus*) compared with an albino mouse.

cAMP content, low amount of vincristine precipitable material in microtubules through which insulin is extruded and low innervation (reviewed in 2,3). These properties were initially attributed to a genetic mutation, which might have occurred during 15 years and 40 generations of maintenance in captivity on fat-rich seed diet. However, it is now most probable that these are typical characteristics of desert animals subsisting on scarce nutrition requiring only limited capacity of insulin response.[4] The low insulin response to glucose and other secretagogues may be a protection against $\beta$-cell overstimulation by sudden availability of nutrients with which spiny mice organism is unable to cope.

A particular characteristic of the spiny mice was the proliferation of $\beta$-cells within islets, accompanying the obesity. The islets increased several fold in number, diameter and $\beta$-cell content (Fig. 4). The resulting very high density of $\beta$-cells in the islets was the highest in comparison with other diabetic obese species (Fig. 5). Thus, spiny mice represent a particular example of obesity associated with enormous enlargement of islets. Among the protective mechanisms which spiny mice were able to mobilize to cope with the increased nutrient intake were increased plasma levels and hepatic production of triiodothyronine ($T_3$) which induced some energy waste and was especially evident on high sucrose diet (Tab. I).[3,5]

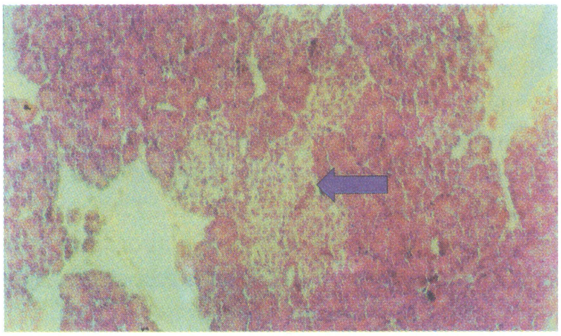

FIGURE 4 Pancreas section of spiny mouse showing hypertrophied islets of Langerhans maintained on fat rich seed diet for 12 months. The arrow points at hypertrophic islets in an irregular shape, filled with $\beta$-cells. Hematoxylin eosin stain, magnification 50x. Adapted from Ref. [96].

### Genetically Endowed Secretion Capacity Determines the Quality of $\beta$-Cells

Other species respond differently to affluent nutrition. Among the responses of $\beta$-cells to

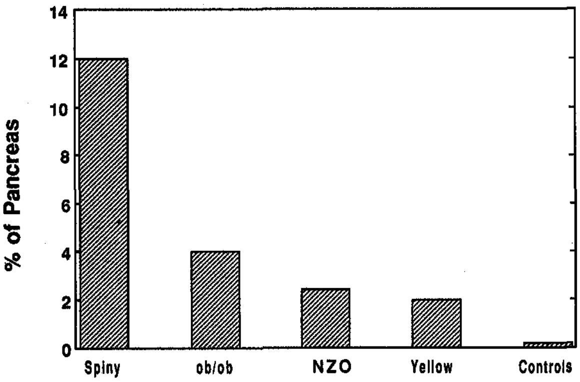

FIGURE 5 Percentage of islets in the pancreas of several obese-diabetic mice compared with an albino mouse as a control. The density of islets in spiny mice is many fold higher than that of *ob/ob*, NZO, yellow KK mice, than of albino mice. Despite high density of islets in the pancreas, the spiny mice are low insulin responders, whereas the other species are insulin oversecretors. Adapted from Gonet *et al.*, *Diabetologia*, 1:162–171, 1965.

TABLE I Effect of sucrose-rich diet on serum $T_3$ and hepatic $T_4 \rightarrow T_3$ conversion in spiny mice

| Spiny mice diets: | Serum $T_3$ ng/ml | Liver $T_3$ ng/liver | $T_4 \rightarrow T_3$ ng/min per liver |
|---|---|---|---|
| Standard | $1.05 \pm 0.09$ | $35 \pm 3$ | $0.95 \pm 0.18$ |
| 50% sucrose | $1.48 \pm 0.10^*$ | $69 \pm 5^*$ | $2.09 \pm 0.28^*$ |

Values are means $\pm$ SE measured after 6 weeks on sucrose diet; *$P<0.01$ at least; Liver wt, g/100g body wt $3.1 \pm 0.2$ standard, $4.6 \pm 0.3$ sucrose.

highenergy (HE) diets we can observe syndromes of diabesity (diabetes + obesity), some of them withstanding and some of them succumbing to the induced insulin oversecretion. There exists a wide spectrum of β-cell response capacity, listed in Table II. Several animal species are endowed with sturdy, resilient β-cells and other with brittle, labile β-cells. Table II indicates that diabetic animals endowed with resilient β-cells exhibit only moderate elevation of blood glucose levels and a capacity of longlasting insulin secretion, whereas those with labile β-cells show transient control of plasma glucose levels by oversecretion, followed by marked hyperglycemia, apoptosis and collapse of β-cells on protracted stimulation of insulin secretion. Hyperinsulinemia in animals endowed with robust β-cells, often associated with hyperphagia, promotes obesity by shunting of nutrients to lipogenesis. Such a shift of glucose to fatty acid synthesis may be looked upon as an antidiabetic measure, moderating the hyperglycemia, which may protect β-cells from glucotoxicity at the expense of obesity.

TABLE II Animals with longlasting *vs.* transient capacity of insulin hypersecretion

| Resilient B cells | Labile B cells |
|---|---|
| C57BL/6J *ob/ob* mice | C57BKS *db/db* mice |
| Zucker *fa/fa* rats | *Psammomys obesus* (sand rat) |
| OLETF rats | |
| WKY rat group | *M. mulatta* (rhesus monkeys) |
| Corpulent *cp* rat strains | |
| KK mice | ZDF/Drt *fa* rats |
| NZO mice | |
| Persistent hyperinsulinemia compensating the insulin resistance lasts for life; high regranulation and mitosis of β cells results in hypersecretion and extremely high insulin levels which shunt glucose to lipogenesis. Thus, hyperglycemia is moderate at the expense of remarkable obesity. Sustained insulin oversecretion reduces FFA release from adipose tissue, preventing β cell lipotoxicity. | β cells transiently proliferate and oversecrete producing a short-lasting weight gain. Then, the degranulation predominates; regeneration and replacement by neogenesis fail to offset the cell loss. There is marked β cell sensitivity to hyperglycemia, intracellular lipotoxicity and detrimental genomic factors. The collapse of the insulin secretion apparatus and apoptosis may be prevented by diet restriction. |

Type 2 diabetes has often been looked upon as a genetic failure of β-cells to compensate for insulin resistance[6] which is true for animals and humans alike. However, this statement does not mean that type 2 diabetes, in humans or animals, is caused by primarily inferior β-cells. Insulin resistant subjects maintain normal glucose tolerance by adaptive hypersecretion of insulin, thereby compensating for the reduction in sensitivity to insulin. In certain individuals these compensatory mechanisms deteriorate with time and overt diabetes supervenes. It is apparent that in these individuals β-cells are genetically not constructed to sustain the demand of oversecretion and are affected by various other genomic factors accentuating the hyperglycemic stress and β-cell exhaustion.[8] Our lesson from animal diabetes, which may be implied for humans, is that insulin resistance constitutes the primary cause of β-cell overtaxation. If the compensatory insulin oversecretion is mild or preventable, even the labile β-cells may last for life.

### Nutritionally Induced Diabetes in *Psammomys obesus*

We have devoted particular attention to the gerbil *Psammomys obesus* (often nicknamed sand rat) The main native nutrient of *Psammomys* is a leafy halophilic plant, *Atriplex halimus*, (saltbush) (Fig. 6). This gerbil never exhibits diabetes in its native desert habitat but was known to develop fatal diabetes when transferred from the Nile Delta in Egypt to the USA.[9] In the 1980s Adler and colleagues have transferred *Psammomys* from the desert shores of the Dead Sea to the laboratory,[10,11] maintaining the animals on low energy (LE) diet, and succeeded to establish a stable, reproducible colony. The animals became diabetic on standard laboratory diet, which is high energy (HE) with respect to *Psammomys* (Fig. 7). The animals are not hyperphagic but when offered their diet ad libitum gradually lapse from normalcy (stage A) into stages of hyperinsulinemia (stage B), hyperinsulinemia with hyperglycemia (stage C), and insulin

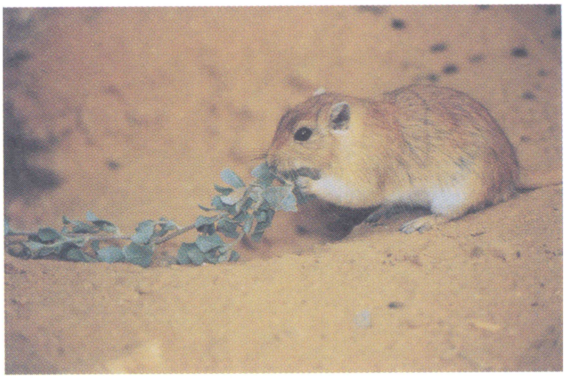

FIGURE 6 Adult *Psammomys obesus* from the Dead Sea region nibbling on salt bush, his native diet. Courtesy of Mr. Barak Negan from Teva Hadvarim magazine.

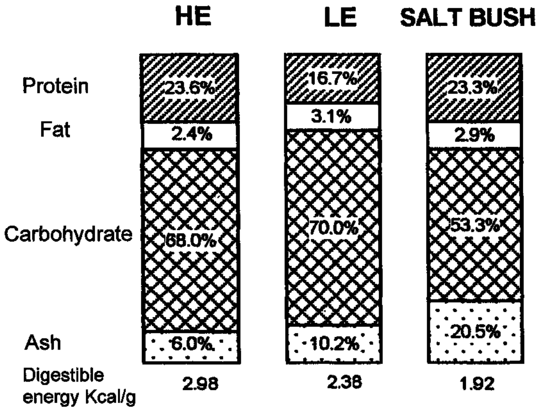

FIGURE 7 Composition of *Psammomys* diets.
LE = low energy diet; HE = high energy diet, about 25% higher in digestible caloric energy mainly of the carbohydrate fraction.

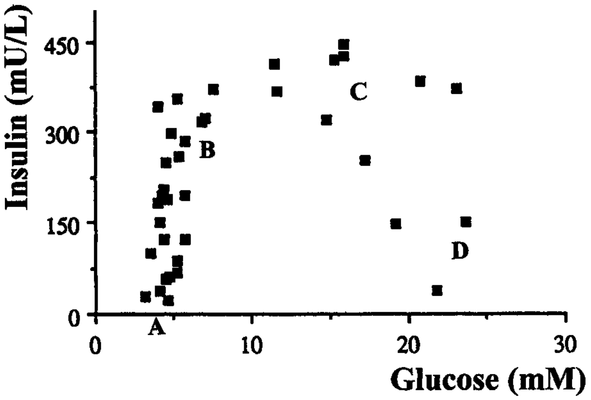

FIGURE 8 Scattergram illustrating the horseshoe pattern of progression of *Psammomys* from normalcy (Stage A) to hyperinsulinemia (Stage B), hyperglycemia (Stage C) and hypoinsulinemia with marked hyperglycemia (stage D). The distribution of glucose and insulin values has been determined in 37, 19 week old *Psammomys* from a general unselected colony. With time most of the animals progressed to stages C and D. Courtesy of Dr. G. Collier, Deakin University, Victoria, Australia.

deficiency with $\beta$-cell apoptosis and necrosis (stage D).[11] The main course of diabetes progress in Israeli *Psammomys* is shown in Figure 8 and described in detail in several publications and reviews.[12–14] Similar observations of the progress to diabetes have been published regarding *Psammomys* from Algeria[15] and a branch of Israeli *Psammomys* colony bred in Australia.[16,17] It should be emphasized that insulin resistance and hyperinsulinemia appear before weight gain in *Psammomys* but they may contribute to adipose tissue accretion, a condition which we term: diabesity. Triglyceride deposition in adipose and nonadipose tissues, primarily muscles is driven by hepatic lipogenesis, which continues unabated despite insulin resistance, as demonstrated in rats.[18] Beacon hypothalamic gene recently discovered by Collier and colleagues[19,20] promotes diabesity on ad libitum feeding. It is remarkable that the progress of *Psammomys* to diabesity may be reversed by reducing the nutrition for just a few days, in stage C, before apoptosis and $\beta$-cell degranulation set in. The recovery by diet restriction has been described in our colony[21] and in *Psammomys* bred in Australia.[22]

*Psammomys* maintained on HE diet for a few weeks undergoes massive $\beta$-cell degranulation, loss of insulin immunostaining, apoptosis and necrosis set in.[23–27] Jörns and colleagues[28] have followed in recent ultrastructural studies the time course of progression of *Psammomys* to diabetes. A gradual loss of $\beta$-cell insulin, glucokinase and GLUT2 transporter immunoreactivities was visualized, occuring subsequently to hyperglycemia. After one week on HE diet the $\beta$-cell volume became reduced by about 1/3 and immunostaining of glucokinase, and GLUT2 by >50% (Fig. 9). After 3 weeks on HE diet this reduction became 70–95% in correlation with the rising blood glucose level. Ultrastructurally

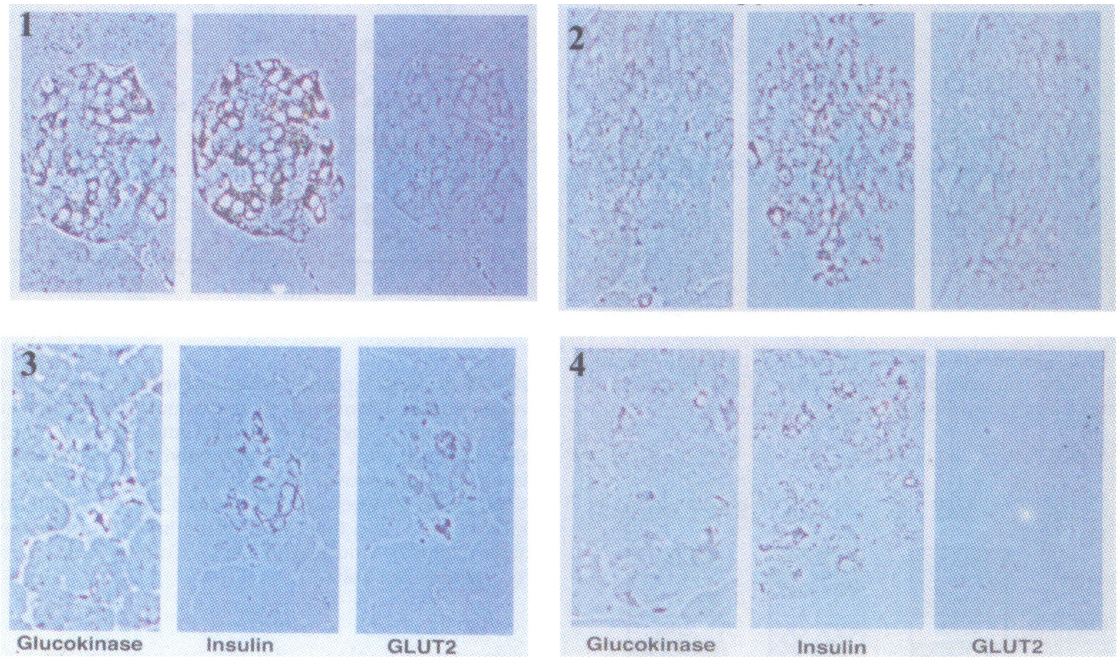

FIGURE 9 Immunocytochemical studies of time course of changes in $\beta$-cells of *Psammomys*. Adapted from Jorns *et al.*, Ref. [28]. **Set 1**: Control pancreas from *Psammomys* on LE diet. Semithin sections stained for insulin, glucokinase (cytoplasm) and GLUT2 transporter (membrane). Magnification 300x; **Set 2**: Hyperinsulinemic *Psammomys* after one week on HE diet. Compared with control pancreas the immunoreactivities of insulin glucokinase and GLUT2 are fainter and moderately reduced. GLUT2 exhibits large gaps. Magnification 400x; **Set 3**: Hyperglycemic, hyperinsulinemic *Psammomys* after 1 week on HE diet. The immunoreactivities of insulin, glucokinase and GLUT2 are markedly decreased compared with nonglycemic hyperinsulinemic animal. Only a few cells exhibited immunostainable insulin. Magnification 500x; **Set 4**: Hyperglycemic *Psammomys* after 3 weeks on HE diet. Extensive $\beta$-cell death. The remaining $\beta$-cells show vacuolization and very faint immunostaining for insulin and glucokinase. GLUT2 is present in the cytoplasm rather than membrane. Magnification 800x.

different signs of necrotic destruction of pancreatic $\beta$-cells such as the pyknosis of nuclei and a massive vacuolization in the cytoplasm were evident. These findings were accompanied by swollen mitochondria and dilated cisternae of the Golgi complex and of the rough endoplasmic reticulum. At one week on the HE diet most secretory granules were still intact even in the face of marked degranulation. Other endocrine cells of the islet did not show structural lesions. These changes in $\beta$-cells were particularly severe in animals after 3 weeks on the HE diet.

The $\beta$-cells in the pancreas removed from hyperglycemic, insulin deficient animals (stage D), after several weeks on HE diet were also found to exhibit apoptosis and DNA cleavage[21,25] (Fig. 10). DNA fragments were seen in the cell nucleus and in the cytoplasm. Also Donath *et al.*[26] and Nesher *et al.*[27] have observed both apoptosis and necrosis in pancreases removed from *Psammomys* after several weeks on HE diet. It is relevant that $\beta$-cell apoptosis was also evident in the diabetic, obese hyperphagic ZDF rats.[29]

Figure 9 clearly shows that the HE diet induced pancreatic $\beta$-cell dysfunction in the *Psammomys* and disintegration of cellular architecture as a consequence of developing hyperglycemia. Hyperinsulinemia by itself does not appear to be responsible for the observed deterioration of the pancreatic $\beta$-cell function, except by the imposed oversecretion. Prolonged incubation of isolated $\beta$-cells from ZDF rats[30] and from humans[31] in high glucose media markedly impaired basal and stimulated insulin secretion. Unger[32] also

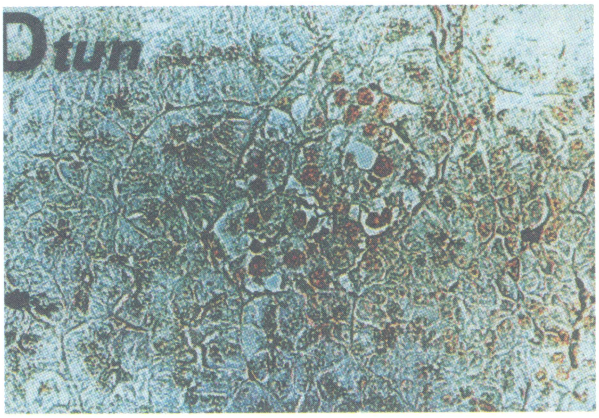

FIGURE 10 β-cell apoptosis in stage D *Psammomys* revealed by Tdt-mediated dUTP nick end labeling (TUNEL) and staining of the biotin labeled DNA cleavage nick ends with 3-aminoethyl carbazole. Note the nuclear fragmentation and spreading of nuclear fragments in the cytoplasm, indicated by the brownish flecks. Magnification 500x. Reproduced with permission of the Editor of *Pancreas*, Ref. [18].

pointed out the deleterious effect of FFA and triglyceride (TG) accumulation in β-cells on the insulin secretion function in ZDF rats, termed by him as "lipotoxicity".

There is no direct evidence for the involvement of gluco- or lipotoxicity in the necrosis of β-cells in *Psammomys* unlike the findings in ZDF rats. An attempt to prevent the possible toxic action of advanced glycation end products or of nitrous oxide by including the advanced glycation inhibitor, aminoguanidine in the hyperglycemic incubation medium was not effective in protecting β-cells in *Psammomys*.[27] However, Kaneto *et al.*[33] obtained evidence that reducing sugars may trigger apoptosis in β-cells of albino rats by provoking oxidative stress of glycation products. In their hands the antioxidants N-Acetyl-L-cysteine and aminoguanidine inhibited the apoptosis. We presume that the damage to β-cell architecture with loss of the insulin biosynthetic and secretory capacity in *Psammomys* occurs promptly and is most probably the result of exhaustion chiefly due to the hypersecretion pressure prior to eventual cytotoxicity.

*Psammomys* in stage C shows increased proinsulin levels in the circulation, up to one half of the circulating immunoassayable total insulin.[34] The inordinate secretion pressure may cause a swift exocytosis of immature insulin granules escaping the C peptide cleavage before the release into the circulation. Similar disproportionate elevation of proinsulin in human and experimental type 2 diabetes and insulin resistance has been observed.[35] This indicates, on one hand, that the compensation of the delayed glucose removal or suppression of gluconeogenesis are not effective since proinsulin has only a minute fraction of insulin activity. On the other hand, the high level of circulating proinsulin does not mean that its secretion equals that of insulin since the half-life of proinsulin is much longer than that of insulin.[36]

### Insulin Resistance and Tyrosine Kinase Attenuation in *Psammomys obesus*

Attenuation of tyrosine kinase (TK) is the basic event responsible for deficient function of the insulin receptor (IR) causing insulin resistance. To investigate the development of insulin resistance, the activity of TK, the initiator of insulin signaling pathway was studied in the liver and muscle of *Psammomys*. Kanety and colleagues[37] found that the binding of insulin to the liver and muscle preparations was very low, even in stage A, indicating the low IR content, about one fifth of the laboratory albino rat. However, insulin binding and TK activity per receptor was completely normal, both *in vitro* and *in vivo*. The TK activity was measured in stages B and C of progression to diabetes as compared to the normoglycemic stage A. Basal phosphorylation of the isolated IR was comparable in these stages to that in the normoglycemic stage A, but the extent of TK activation by insulin was markedly lower in stages B and C in the liver and muscle (Fig. 11).[37] The reduced insulin activation was accompanied by a marked decrease in muscle GLUT4 protein and mRNA (Fig. 12). Both could be reversed by nutritional restriction to one half of their daily food intake for a few days. The recovery of TK activity was complete when the animals returned to normoinsulinemia. The recovery was partial when hyperglycemia was corrected but the insulin

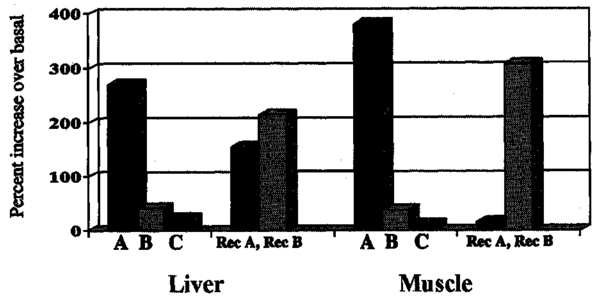

FIGURE 11 Activation of tyrosine kinase in isolated insulin receptors from muscle and liver of *Psammomys* at stages A, B, and C. Stages B and C are compared to stage A and the full or partial recovery on diet restriction is marked Rec A and Rec B. Receptors were purified on wheat-germ agglutinin and the phosphorylation of poly(glu : tyr)4 : 1 substrate in the presence of 50 μM [$\gamma^{32}$P]ATP was determined before and after stimulation with insulin and expressed as percentage change of the basal activity. Adapted from data of Kanety *et al.*, Ref. [37].

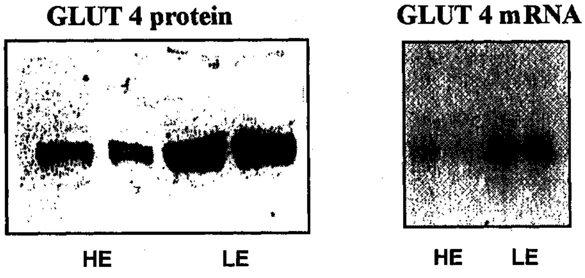

FIGURE 12 Western blot of membranal GLUT4 protein and mRNA in gastrocnemius muscle of *Psammomys* on HE and LE diets. Densitometry indicated a mean decrease in stage C *Psammomys* to 40% (protein) and 20% (mRNA) of values in stage A. Adapted from Ref. [50].

levels did not return to normal (Fig. 11). This finding points out the attenuating effect of hyperinsulinemia on the function of the IR, indicating that it is an important cause of insulin resistance.

### Deleterious Effect of Hyperinsulinemia on IR Function

The deleterious effect of hyperinsulinemia, even in non-nutritionally induced conditions, can be demonstrated in several animal species and humans. A few cogent examples can be quoted. Transgenic mice enriched with 8 or 32 insulin gene copies, resulting in circulating hyperinsulinemia, exhibited both IGT and hypertriglyceridemia in correlation with the amount of insulin gene copies in their $\beta$-cells (Fig. 13).[38] In other transgenic mice insulin oversecretion was related to deleterious overexpression of glutamine : fructose 6 phosphate amidotransferase associated with $\beta$-cell malfunction.[39] Hyperinsulinemia and insulin resistance was also achieved by targeted disruption of genes

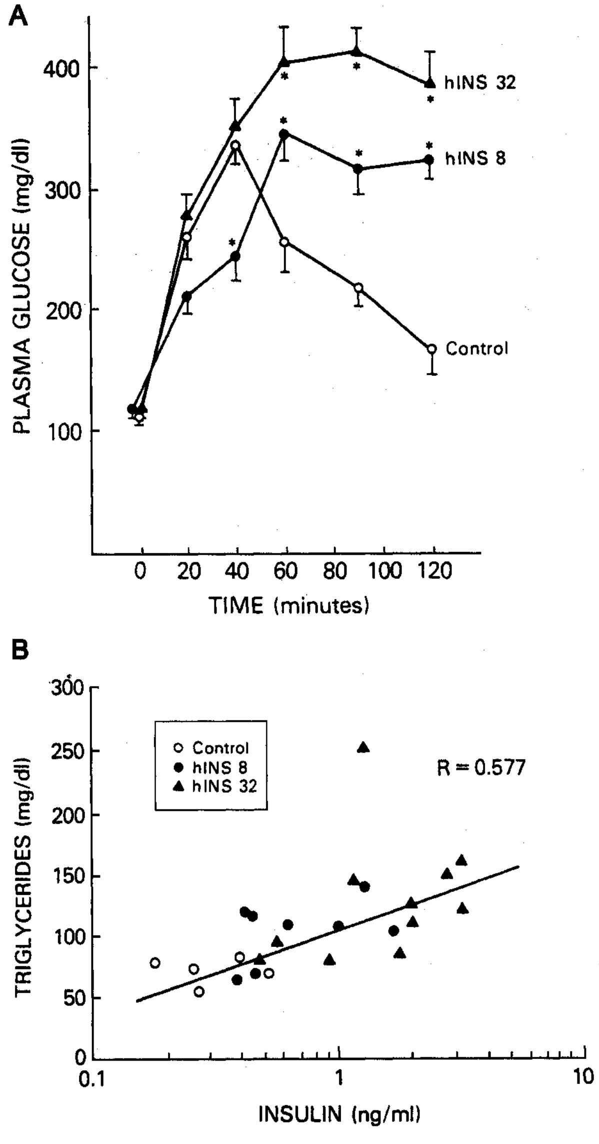

FIGURE 13 **A.** Intraperitoneal glucose tolerance (2 mg glucose/g) in transgenic mice transfected by pronuclear microinjection with 8 or 32 copies of human insulin gene. Age 10–12 months, mean ± SE of 9 determinations. Controls were non-transgenic litter mates. **B.** Correlation between rising plasma insulin and triglyceride levels in transgenic mice. O control. ● 8 gene copies. ▲ 32 copies of insulin gene. $r = 0.577$, $P < 0.001$. Adapted from S. L. Marban and J. Roth, *Lessons from Animal Diabetes* 5, 201–204, 1996.

encoding the insulin signaling and glucose transport molecules in mice, *e.g.*, IRS and GLUT4 as recently reviewed by Sone *et al.*[40]

Miles *et al.*[41] have show that protecting the insulin from hepatic degradation by diversion of pancreatic blood flow through anastomosis with vena cava in healthy dogs induced a sustained hyperinsulinemia. The hyperinsulinemia resulted in marked insulin resistance evident from the 30% reduction in peripheral glucose disposal rate. The resistance was localized to IR/TK, the maximal activation of which by insulin decreased to $12.7 \pm 1.7$ *vs.* $20.3 \pm 2.7$ fmol phosphate/fmol IR in control dogs, a reduction of about 40%.

Among other detrimental results of hyperinsulinemia is the uncoupling of the TK activity in 3T3 cells after initial activation.[42] In HepG2 cells the activation of IR TK by insulin was attenuated; only incompetent receptors remained on cell surface[43] and in rat adipocytes the $V_{max}$ of TK was reduced.[44] It was also found that hyperinsulinemia inhibits myocardial protein degradation in patients with cardiovascular disease, which is a potential mechanism contributing to cardiomegaly.[45] Hyperinsulinemia of endogenous or exogenous origin should be considered, also in humans, not only as a compensatory response to insulin resistance, but as an inducer of a defect in insulin action. In nondiabetic human volunteers the infusion of insulin for several days followed by euglycemic hyperinsulinemic clamp, resulted in the reduction of nonoxidative whole-body glucose metabolism by up to 40%.[46] Also, patients with insulinoma exhibited insulin resistance that was related to the extent of their hyperinsulinemia.[47] Furthermore, fasting hyperinsulinemia in diabetes-prone Pima Indians has been found to exert a primary role in the progress to type 2 diabetes by being the antecedent of the decline in response to i.v. glucose load.[48]

### Overexpression of PKC – A Negative Feedback in Insulin Signal Transduction

Protein kinase C (PKC) in the gastrocnemius muscle of *Psammomys* was found pronouncedly overexpressed.[49,50] This enzyme group has now become widely studied because of their preferential phosphorylation of serine and threonine residues on cellular proteins, thus leading to the malfunction of IR and of other proteins active in this pathway.[51] The PKC group includes at least 11 isoforms, among them, the so-called conventional PKC$\alpha$, $\beta$1, $\beta$2 and $\gamma$, which are DAG sensitive. The novel isoforms, PKC $\delta$ $\varepsilon$ $\eta$ $\theta$ are also DAG sensitive. The atypical forms $\zeta$ and $\lambda$ are DAG insensitive. Some of these isoenzymes have been termed "lipid second messengers" because of their DAG dependence.[52] Attribution of a specific function to an PKC isoenzyme has not been yet firmly established. Several isoenzymes forms may mediate a similar range of functions. Among the several PKC isoenzymes probed with specific antibodies PKC$\varepsilon$ was most significantly overexpressed in the skeletal muscle of *Psammomys*, in the hyperglycemic-hyperinsulinemic stage C compared with the nondiabetic stage A (Fig. 14). It was also translocated from the cytosol to muscle membrane to a larger extent than other PKC isoenzymes, which indicates not only PKC$\varepsilon$ overexpression but increased activity as well.[49] As shown in Table III about 1/3 of total PKC$\varepsilon$ cell content resides in the membrane fraction. PKC$\theta$

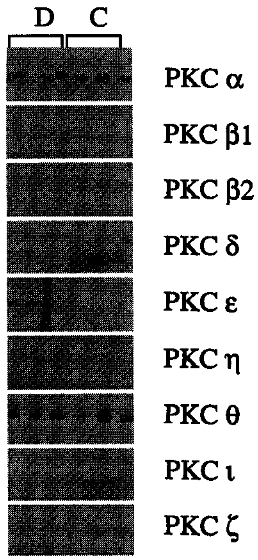

FIGURE 14 Immunoblots showing the membranal content of PKC isoenzymes in gastrocnemius muscle of *Psammomys obesus*. Note the overexpression of PKC$\varepsilon$. C = control, D = diabetic stage C. Courtesy of Dr. Luitgard Mosthaf-Seedorf.

TABLE III Subcellular distribution of PKC isoenzymes in *Psammomys* muscle: membrane/total (%)

| | DR | DP/A | DP/C |
|---|---|---|---|
| PKC$\alpha$ | 26.1 ± 5.9 | 31.6 ± 4.1 | 39.6 ± 1.7* |
| PKC$\beta_I$ | 12.8 ± 2.0 | 17.7 ± 2.6 | 20.8 ± 1.3** |
| PKC$\beta_{II}$ | 26.5 ± 3.7 | 32.1 ± 6.8 | 34.7 ± 3.1 |
| PKC$\varepsilon$ | 26.7 ± 1.6 | 27.5 ± 1.5 | 33.5 ± 0.7†# |
| PKC$\theta$ | 72.9 ± 2.4 | 72.9 ± 4.0 | 65.5 ± 1.9 |
| PKC$\eta$ | 16.4 ± 3.1 | 16.1 ± 4.9 | 18.8 ± 1.6 |
| PKC$\zeta$ | 35.8 ± 5.0 | 34.4 ± 6.0 | 39.6 ± 3.4 |

Values are means ± SE; *DP/C *vs.* DR P<0.005; **P<0.01; †P<0.001; #P (DPC *vs.* DPA)<0.01. Reproduced from Ikeda *et al.*[49] with permission.

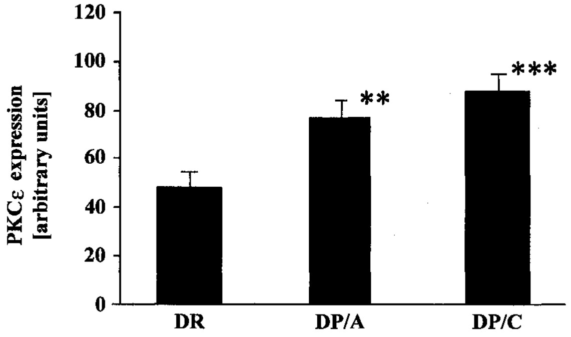

FIGURE 15 Overexpression of PKC$\varepsilon$ in stages A and C of *Psammomys* muscle compared to the diabetes resistant line (DR). Note a significant overexpression of PKC$\varepsilon$ already in normoglycemic-normoinsulinemic stage A of diabetes-prone line of *Psammomys* indicating the innate insulin resistance preceding the onset of diabetes, without consequences on the LE diet.

showed highest degree of membrane association in Stage A *Psammomys* but was surprisingly low in Stage C. The membranal PKC$\alpha$ and $\beta$ were also elevated. PKC$\zeta$ was elevated but did not change between stages A and C. PKC $\gamma$ and $\zeta$ (the atypical isoenzymes) are known to promote phosphorylations integral to the insulin signal transduction.

We have compared the expression of several PKC isoenzymes in diabetes resistant (DR) and diabetes prone (DP) *Psammomys* lines. The DR line was isolated from the parent *Psammomys* colony by assortative mating of individuals, which did not exhibit hyperglycemia and hyperinsulinemia on HE diet.[53] It was found that the difference between the DR and DP animals is related to the efficiency of nutrient utilization: the cost of weight gain upon growth is in *Psammomys* DR = 9.3 kcal/g and DP = 6.0 kcal/g. Interestingly, a significant overexpression of PKC$\varepsilon$ was also observed in the normoglycemic stage A of DP Psammomys compared with the DR line (Fig. 15), which indicates that PKC$\varepsilon$ overexpression precedes the onset of overt insulin resistance. Thus, PKC$\varepsilon$ overexpression in stage A may be considered as a marker of "prediabetic" or "preinsulinemic" stage and of propensity of a given individual to progress to overt diabetes on affluent nutrition. It is, however, without untoward consequences as long as the diet is LE.

Additional evidence of the innate insulin resistance in stage A *Psammomys* was demonstrated *by* the failure of external insulin administration to effect hypoglycemia. Insulin also failed to suppress in stage A *Psammomys* the activity of hepatic PEPCK, the rate limiting enzyme of gluconeogenesis,[54] as well as the hepatic glucose output (Fig. 16). This may be a typical characteristic

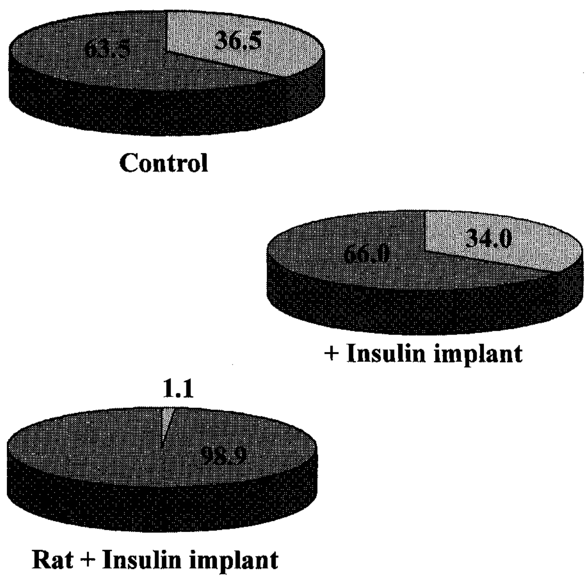

FIGURE 16 Hepatic glucose output (HGO) in *Psammomys* stage A after s.c. administration of insulin implants releasing 2 U insulin/24 h with glucose levels reaching about 300 mU/l at 4 hours. The white section of the circle denotes the non-suppressed HGO. Note the inability of insulin to suppress the HGO respective to control stage A *Psammomys*, whereas it almost completely shuts off the glucose output in the albino rat and renders the rat deeply hypoglycemic. The activity of hepatic phosphoenolpyruvate kinase was also not reduced by the insulin, confirming the inherent insulin resistance in stage A *Psammomys*. Based on Ref. [54].

of a desert animal in which muscle insulin resistance saves glucose for the support of other glucose obligatory tissues.

Since PKC$\varepsilon$ overexpression resulted in impaired TK activation by insulin and reduced GLUT4 mRNA and protein, which indicates an impaired PI3K activation, it was of interest to investigate whether PKC overexpression induces a further negative downstream defect in insulin signaling. The activity of PKB/Akt was determined, an enzyme regarded as responsible for the activation of pleiotropic metabolic systems within the cell, subsequent to PI3K activakon on IRS. The transfection of HEK 293 cells with IR and/or PKC$\varepsilon$ plasmids, followed by stimulation with insulin or TPA respectively, clearly showed the activation of PKC$\varepsilon$ by TPA coupled with a significant reduction of PKB expression and inhibition of PKB activation (Fig. 17). These results indicate that PKC$\varepsilon$ inhibits PKB activation by insulin and has a far-reaching negative effect on metabolic reactions dependent on insulin signaling as illustrated in the insulin signaling scheme (Fig. 18).

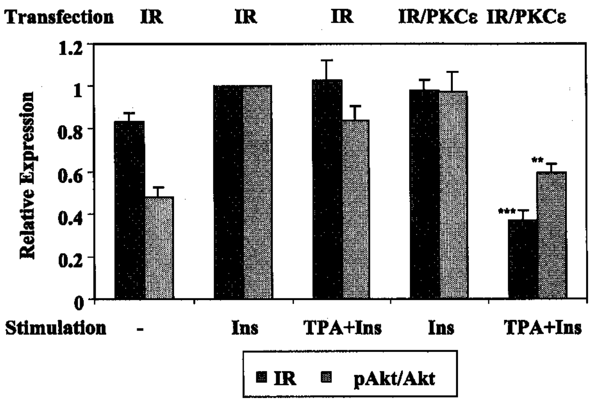

FIGURE 17 Attenuation of protein kinase B (PKB) by contact with PKC$\varepsilon$. Human embryonic kidney cells (HEK293) were transfected with IR and PKC$\varepsilon$ expression plasmids together or alone and incubated overnight in medium containing 0.5% fetal calf serum. The cells were left untreated or stimulated with $10^{-7}$mol/l insulin for 5 min or treated with 1 $\mu$mol/l TPA for 6h before insulin stimulation. Cell lysates were subjected to SDS-PAGE, and incubated with specific antibodies against IR $\beta$-subunit, Akt, or phospho-Akt (Ser 473). Proteins were made visible using horseradish-peroxidase-coupled secondary antibodies and chemiluminescence. The diagram shows means ± SD of three independent experiments. The data are normalized to the expression of cells transfected with IR alone and stimulated with insulin. From Ikeda *et al.*, Ref. [49] (with permission).

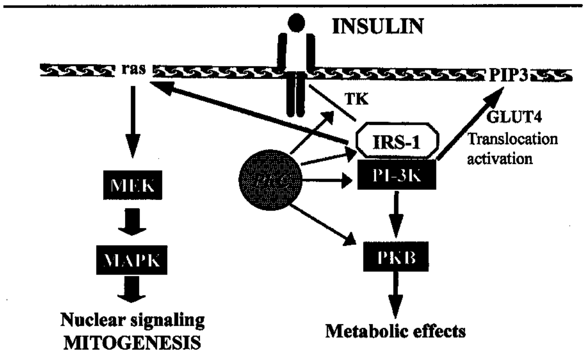

FIGURE 18 Insulin signaling scheme pointing to the PKC$\varepsilon$ effects on tyrosine kinase, PI3K and PKB activities. The latter is responsible for activation of multiple metabolic systems.

The activity of PKC isoenzymes in the membrane, in IR proximity, may be the reason for the inhibitory influence on the IR TK activation. Several PKC isoenzymes were reported to reduce the TK catalyzed phosphorylation of the IR $\beta$-subunit and IRS-1.[55–60] We have found that PKC$\varepsilon$ overexpression was associated with reduced binding of insulin by muscle IR (Fig. 19). This was not likely to be attributed to the impaired binding capacity of IR but to the reduction in the number of IR per cell. Indeed, the downregulation of IR was demonstrated in HEK 293 cells which were transfected with human IR and PKC$\varepsilon$ plasmids and activated by TPA (Fig. 20). Evaluation by densitometry showed that

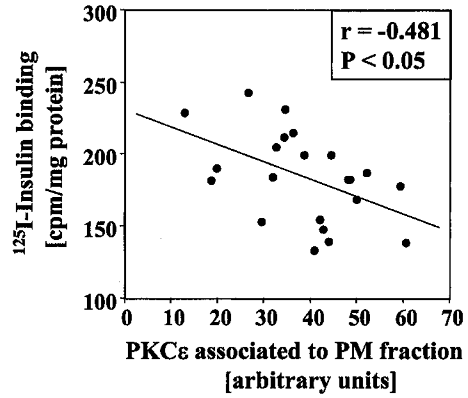

FIGURE 19 Reduced insulin binding by muscle of *Psammomys* in stage C measured by ELISA assay. Linear regression analysis of PKC$\varepsilon$ associated to plasma membrane fraction *versus* $^{125}$I-insulin binding in muscle homogenates. n = 22, P < −0.05. From Ref. [49] with permission.

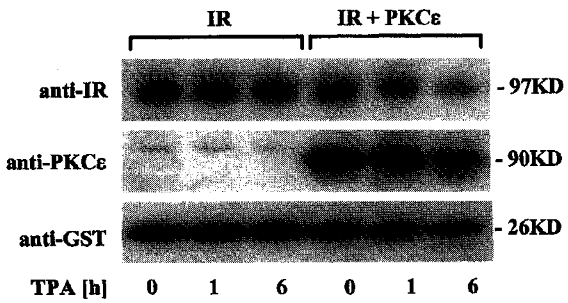

FIGURE 20 Insulin receptor degradation effected by PKCε overexpression. HEK 293 cells were transfected with IR and PKCε expression plasmids either alone or in combination and incubated overnight in a medium containing 0.5% fetal calf serum as a control. Glutamine S transferase construct was co-transfected in all samples. The cells were treated with 1 mmol/l TPA as indicated. Cell lysates were then subjected to SDS-PAGE and incubated with anti-IR-specific antibodies. Proteins were visualized using horseradish peroxidase-coupled secondary antibodies and chemiluminescence. The upper rows show PKCε expression with and without IR. The lower rows GST expression levels as control. Quantitation as percentage of control has been performed in 6 independent experiments showing a decrease in the IR content in the presence of TPA-activated PKCε, to 30% of control. $P > 0.001$ *vs.* both IR alone and IR + PKCε unstimulated samples. Reproduced from Ikeda *et al.*, Ref. [49] (with permission).

after 6h of TPA activation the amount of IR was reduced to about 40% of the original number. This is in accord with previous observations of downregulation of IR, by the conventional PKCα[61] and possibly other DAG sensitive PKC enzymes.

Several *in vitro* studies indicate that PKC isoenzymes directly interfere with insulin signaling through serine/threonine phosphorylation of either the IR itself or one of its major substrates.[62–64] PKC may also mediate the tumor necrosis factor (TNFα) inhibition of IR function, the major cause of diabesity-linked insulin resistance.[65,66] TNFα was reported to induce phosphorylation of IRS-1 on serine 307.[67] Interestingly, high insulin levels also induced the phosphorylation of this serine in association with insulin resistance in signal transduction. This observation suggests the possibility of PKC involvement. Several other serine sites have been indicated to be phosphorylated on IR or IRS with negative effects on signal transduction.[68–70] Muscle PKC activation was also seen in insulin resistant Goto-Kakizaki rats.[71] It may be therefore assumed that serine/threonine phosphorylation of IRS-1, inhibits the TK activity of the IR *via* a feedback loop and is responsible for the deficient TK activation by insulin in *Psammomys* as described earlier,[37] with insulin resistance accentuated at stages B and C on the HE diet.

### PKCε Overexpression and Muscle Lipid Content

The enhanced PKCε activity and/or expression in *Psammomys* was found to be correlated with the increased muscle content of DAG (Fig. 21).[49] DAG is an intermediate of both fatty acid esterification to TG and TG breakdown to fatty acids and glycerol. The raised muscle concentration of DAG results from increased TG deposition and turnover in muscle, which occurs in the situation of hyperinsulinemia and hyperglycemia, characteristic of stages B and C of *Psammomys*. An increase in incorporation of glucose carbon into DAG was also seen in the soleus muscle incubated with glucose and insulin[72] probably in relation to increased intracellular TG synthesis. Also, the rise in plasma FFA in conditions of IGT may contribute to muscle fat deposition. Indeed, *in vitro* uptake of saturated FFA was recently reported by Yu *et al.*[73] to raise rat muscle DAG levels and lead to PKC activation (Fig. 22). Exogenous lipid infusion in rats resulted in the

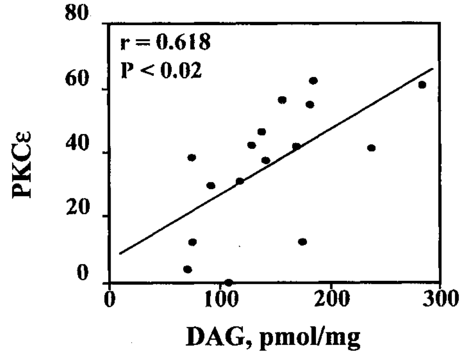

FIGURE 21 PKCε activity correlated to intracellular content of diacylglycerol (DAG) in the gastrocnemius muscle of *Psammomys*. Note a high correlation coefficient of both membrane associated PKCε and PCKα with muscle DAG content. Among other PKC isoenzymes only PKCα showed a similarly significant inverse relationship. Reproduced from Ikeda *et al.*, Ref. [49] (with permission).

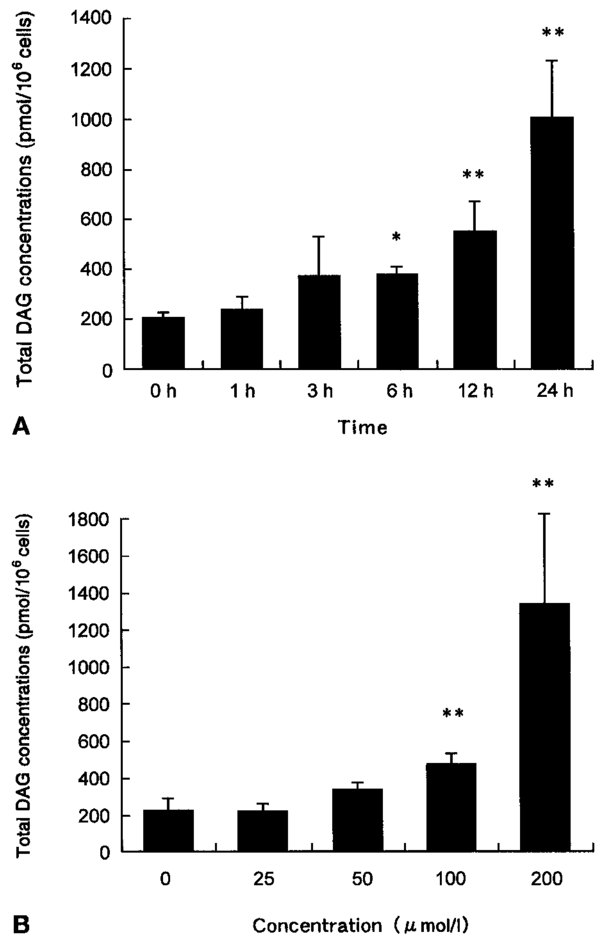

FIGURE 22 FFA uptake and muscle DAG content. Time (A) and concentration (B) dependent increase in diacylglycerol (DAG) in cultured smooth muscle cells incubated with palmitate. Results are means ± SE from 3 separate experiments in triplicate normalized by cell number. Asterisks denote significance at $P < 0.05$ and $< 0.01$ compared to the basal DAG value. Reproduced from Ref. [71] with permission of Diabetologia, Springer Verlag.

deposition of a significant fraction of fatty acids in muscle, particularly in the fasted state[74] and FFA infusion to human subjects was found to elicit insulin resistance and activation of PKC$\theta$.[75] Muscle TG content is also increased in patients with type 1 diabetes although it did not correlate in this condition with insulin sensitivity.[76]

Increased TG deposition in muscles was studied extensively by Kraegen and colleagues[77–79] in rats maintained on a fat-rich diet. Insulin resistance developed in these rats both in muscles and liver. The hepatic insulin resistance was associated with increased gluconeogenesis, whereas the muscle insulin resistance markedly reduced the insulin stimulated glucose uptake. The accumulation of muscle fat was inversely correlated with insulin resistance and delayed glucose disposal also in human subjects.[80] Schmitz-Pfeiffer *et al.*[80] found that in high fat fed rats, TG and DAG accumulated in muscle and activated PKC isoenzymes interfering with IR function. The total expression of PKC $\alpha$, $\varepsilon$ and $\zeta$ isoenzymes was not increased in muscles of these rats but their activity and distribution between cytosolic and membrane compartments was shifted in favor of the latter. This was particularly prominent in the case of PKC$\varepsilon$ showing a sixfold increase in the membrane/cytosol ratio in correlation with muscle TG content (Fig. 23). There was no accumulation of TG and DAG in control rats fed a starch diet. Also in *Psammomys* the muscle and liver TG content increased on HE diet but the increase was moderate in comparison with fat-fed rats (Fig. 24).[14] This does not necessarily mean that muscle lipid deposition is a result of outright obesity, but even a small weight gain usually occurring prior to marked hyperglycemia in *Psammomys* leads to TG deposition, also in nonadipose tissues.

### Protein Tyrosine Phosphatases and Muscle Insulin Resistance in *Psammomys*

Goldstein and colleagues[81–83] have reviewed the mode of action of PTPases and their impact on the regulation of IR signaling by modulating the tyrosine phosphorylation state of IR and of proteins that transmit the insulin signal. PTP1B is considered to play a key role in glucose homeostasis and energy expenditure and has been pointed out as an important negative regulator of insulin action.[83–85] Mouse transgenic and knockout models with altered expression of LAR (Leukocyte Antigen Related), PTP1B and SHP-2 PTPases generated additional insight into the involvement of these regulatory enzymes in insulin action and glucose metabolism. LAR PTPase can have a negative impact on cellular insulin signaling, although its exact physiological role has not

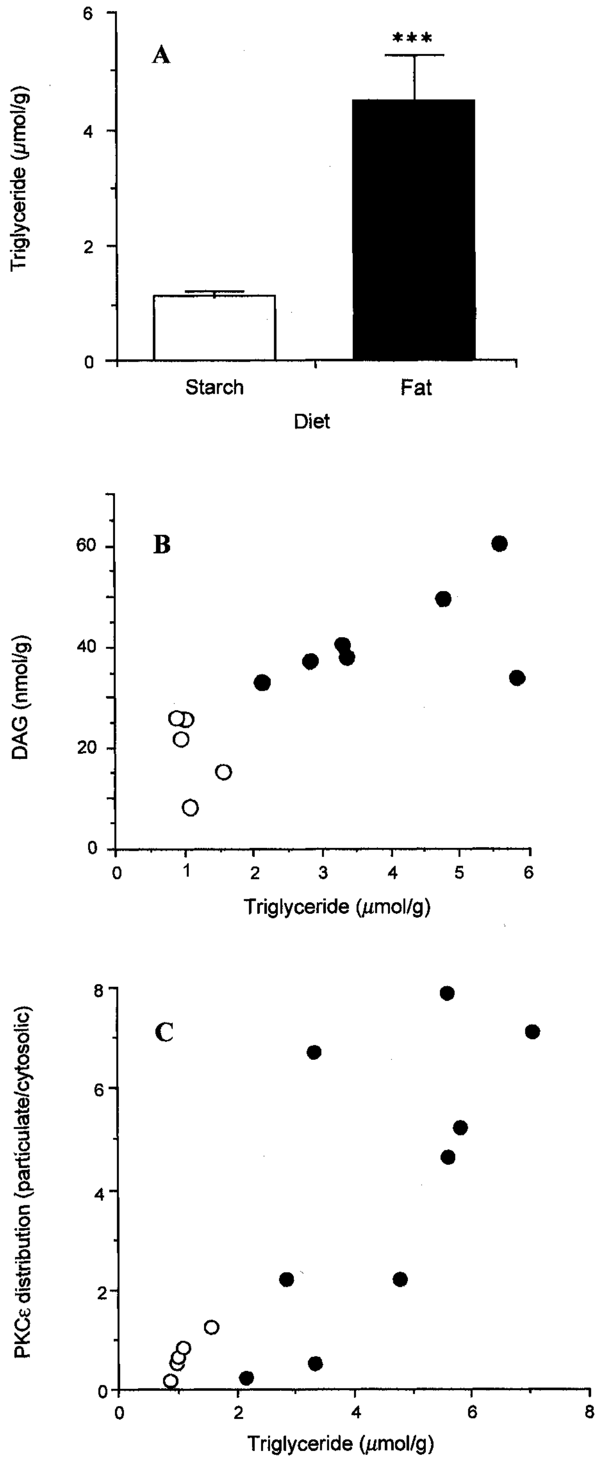

FIGURE 23 **A.** Relation of gastrocnemius muscle triglyceride and DAG contents in rats fed fat-rich and starch diets. **B.** Total PKCε expression was not increased but the PKCε membranal/cytosolic ratio to DAG content was pronouncedly elevated. Adapted from Schmitz-Pfeiffer *et al.*, Ref. [79].

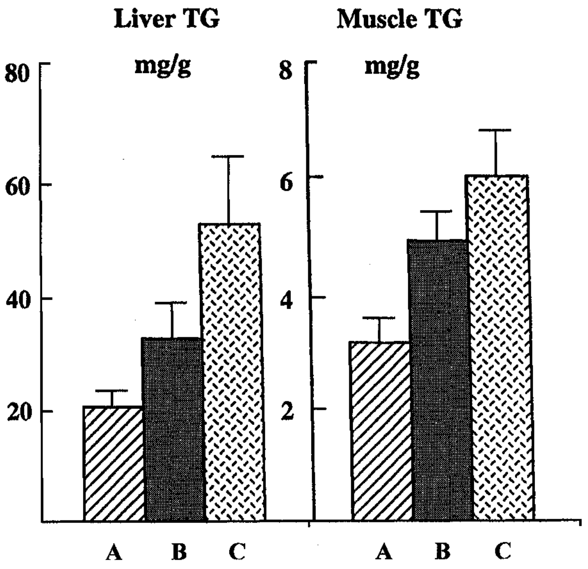

FIGURE 24 Tissue triglyceride content in *Psammomys*. Liver and muscle triglyceride levels in *Psammomys* in stages A–C. Values are mean±SE for groups of 10–14 animals at each stage differences significant at P<0.05 at least. Adapted from Ref. [14].

been established. On the other hand, SHP-2 positively influences receptor signaling on mitogenic pathways in cellular studies. The reduction of tyrosine phosphorylation effected by TK activity could be caused by enhanced dephosphorylation of the receptor β-subunit and IRS-1, carried out by the action of the tandem domain transmembrane LAR PTPase. Abundance of LAR-PTPase was observed in skeletal muscle and liver of rodents with genetically determined insulin resistance rats and in human obese patients.[86,89]

The activity and expression of LAR PTPase was investigated in *Psammomys*.[90] In stage A, a low PTPase activity in liver and muscle was found, in parallel with the low density of insulin receptors. Fasting caused a decrease rather than increase in LAR-PTPase in stage A *Psammomys* (Fig. 25). However, *Psammomys* tissues in stage C did not show an increase in cytosolic or membranal LAR PTPase activity, compared with stage A, suggesting that LAR-PTPase is unlikely to be responsible for IR dephosphorylation in insulin resistant *Psammomys*. This observation parallels the findings in human nutritionally induced diabetes. Molecular and linkage analysis of type 1 PTPase

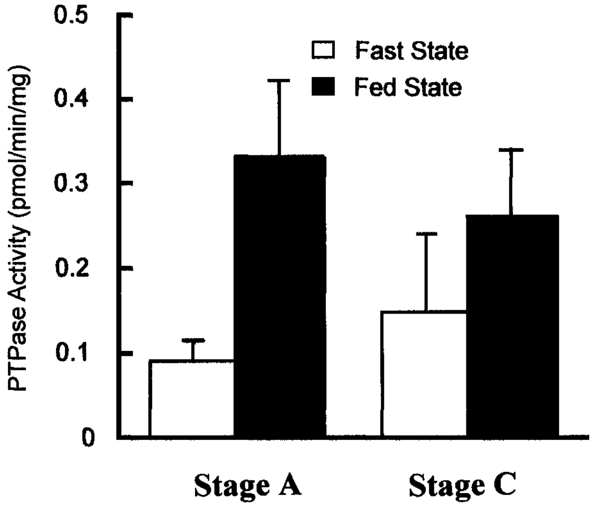

FIGURE 25 LAR PTPase activity in *Psammomys* muscle. Cytosolic LAR PTPase activity on HE diet (stage C) showing a tendency to increase after overnight fasting but a decrease in the fed state. Basal activity in the fed and fasted state is not significantly different.

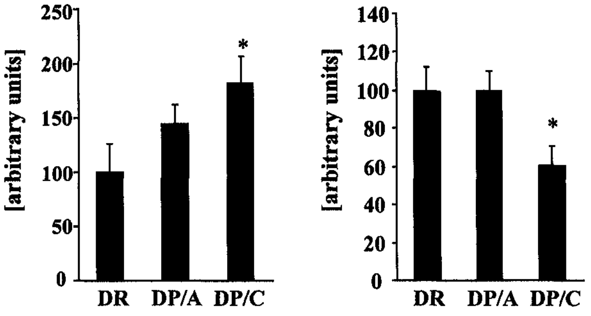

FIGURE 26 PTP1B expression (left side) and activity (right side) in *Psammomys* muscle. The expression of PTP1B was increased in stages A and C on HE diet compared to DR *Psammomys* set to 100. However, the activity of the enzyme measured by dephosphorylation of isolated IR decreased in stage C probably as a result of enzyme impairment in the diabetic milieu. Mean±SE of 10 samples $P<0.005$ *vs.* DR. From Ikeda *et al.*, Ref. [92].

on the $\beta$-subunit gene was not consistent with a role in insulin resistance in Pima Indians.[91]

To examine whether PTP 1B is involved in the susceptibility to insulin resistance or diabetes in *Psammomys*, Ikeda *et al.*[92] have measured its expression and activity towards the isolated IR of skeletal muscle of diabetic animals from DP line and control animals from the DR line. The expression level of PTP 1B in the skeletal muscle was increased by 83% in the diabetic animals compared with the control animals. However, when the activity of PTP 1B was determined there was a surprising 60% decrease in its activity in stage C *Psammomys*. This was seen in the total homogenate and especially in the particulate fraction when compared with the control DR animals or with the prediabetic animals (Fig. 26). In addition, PTP 1B activity was inversely correlated to serum glucose concentrations and insulin levels. The decrease in activity was assumed to be due to qualitative change in the PTP 1B molecule, probably secondary to the effect of some factor(s) in the diabetic milieu. These findings suggest that PTP 1B is not involved in the development of insulin resistance in *Psammomys*. The overexpression of PTP 1B in skeletal muscle does not appear to be genetically determined but may represent a compensatory mechanism against such impairment of the enzyme. Thus, the likely conclusion emerges that there is no evidence for negative regulatory influence of PTPases in the nutritionally induced diabetes of *Psammomys*. Support for this contention comes from the findings of Worm *et al.*[93] that the skeletal muscle PTPase in insulin resistant Zucker *fa/fa* rats was found to be downregulated. The decrease in its activity could be prevented by normalizing glucose and insulin levels by treatment with metformin.

### Conclusions and Overview

*Psammomys* is a model of human nutritionally induced diabetes which reaches now epidemic proportions in certain populations. The underlying cause is increased food availability and consumption following a welcome improvement in lifestyle. However, the latent propensity to diabetes among these populations is related to their inborn metabolic capacity which is probably not adjustable to the dietary surplus. This may result in insulin resistance, diabesity and strain on compensatory insulin secretion with ultimate loss of $\beta$-cell function. Some authors refer to such socioeconomic perspective as leading to nutritional genocide of global proportions. The above described elements of insulin resistance in *Psammomys* represent the antecedents of the development of worldwide diabetes epidemic in human populations emerging from food scarcity to food abundance.

Insulin resistance and $\beta$-cell dysfunction are considered two interrelated factors in the pathogenesis of IGT and type 2 diabetes. Although a debate is still continuing on the primacy of each abnormality, the evidence from *Psammomys* studies clearly demonstrates that insulin resistance with hyperinsulinemia precede the $\beta$-cell lesion. $\beta$-cell dysfunction occurs only on HE diet, there is no $\beta$-cell lesion in animals consuming their native salt bush or laboratory LE diets. The onset of insulin resistance and hyperinsulinemia in *Psammomys* precede any appreciable weight gain, precluding any contribution of obesity to IGT. On the contrary, if hyperinsulinemia with $\beta$-cells oversecrete long enough the expansion of adipose tissue may occur and secondarily lead to overweight. A return to normalcy is possible even after a period on HE diet, either by a short term fasting, or by restricting the dietary intake. It is most probable that similar triggering of IGT and diabetes applies to the affected human populations.

The aberrant activity of PKC isoenzymes, especially of PKC$\varepsilon$, is the potential causative mechanism in the generation of insulin resistance by phosphorylation of serine/threonine residues on IR and proteins of the signaling pathway. This may lead to TK, PI3K and PKB attenuation with negative feedback as well as to IR degradation. Thus, the compensatory hyperinsulinemia precludes the adequate function of insulin signaling.

One of the primary outcomes of the overexpression of PKC isoenzymes may include PKC$\beta$, involved in the initiation of vascular complications of diabetes in insulin independent tissues as retina and kidneys.[94,95] The common aspect of this overexpression with *Psammomys* and fat-fed rats is tissue accumulation of DAG. DAG is directly related to tissue TG content and this may be an especially important inducer of insulin resistance in nonadipose tissues. Insulin resistance and its corollaries may then result from enhanced muscle lipid deposition, not necessarily from hyperlipidemia. The initial fat deposition may be also promoted by hyperinsulinemia with hyperglycemia and the following diabesity.

The preventive attempts should be therefore directed to avoiding muscle TG deposition, promotion of DAG breakdown by use of modalities activating DAG kinase, which converts DAG into phosphatidic acid. Other possibility to counteract the insulin resistance is specific inhibition of PKC isoenzymes dependent on DAG. Among those is H7 – a piperazine derivative, polymyxin B, bisindoxylmaleimide and staurosporine which are inhibitory to PKC isoenzymes *in vitro*.[96] Herbimycin was also shown to have PKC inhibitory properties.[97] Inhibition of PKC$\beta$ with high degree of specificity has been achieved by LY 33353[98] and tried successfully in vascular tissues, mainly ocular and renal, which are predisposed to complications in hyperinsulinemic-hyperglycemic conditions. It is also remarkable that the recently reported PKC$\theta$ knockout in mice improved insulin action and signaling defects induced by lipid infusion.[99]

Potentiating insulin sensitivity at its prevalent concentrations would also lead to lowering of insulin resistance as shown by the application of IR activators.[100] Increased insulin sensitivity was also achieved by treatment with vanadyl sulfate and other vanadium compounds[101–104] including *Psammomys* maintained on HE diet (Fig. 27). Vanadyl sulfate restoration of normoglycemia and normoinsulinemia and increase in muscle metabolic activity appears to be distal to IR/TK signaling.[101]

The overexpression of PKC isoenzymes may be the result of genetic susceptibility exemplified by *Psammomys* or by "thrifty gene" characteristics of desert animals or individuals in the affected populations, activated by the changing environmental influences. This course of events is illustrated by Figure 28. The inherent muscle insulin resistance aimed to spare the scarce glucose for obligatory tissues (such as the brain) fails when confronted with excess of nutrients. It turns the insulin resistance to effect a misuse of the surplus energy by creating diabesity, hyperlipidemia and elicit other complications. Such situation is most probably an integral component

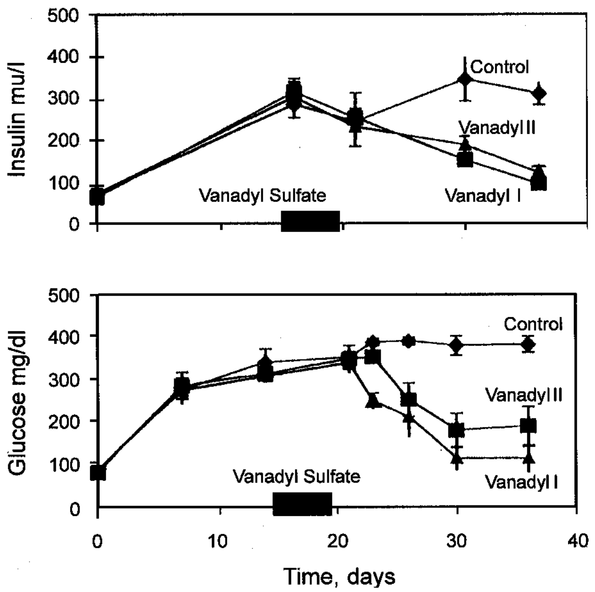

FIGURE 27 Vanadyl potentiation of insulin action. *Psammomys* on HE diet was treated i.m. with vanadyl sulfate (5 mg/kg for 5 days), after maximal glucose and insulin levels in plasma were reached (stage C). Vanadyl I: complete recovery to control glucose and insulin levels in 10 of 17 animals. Vanadyl II: Marked but incomplete recovery in 7 of 17 animals. The low levels of glucose and insulin persisted for at least 15 days after vanadyl administration. Reproduced from Ref. [100] with permission.

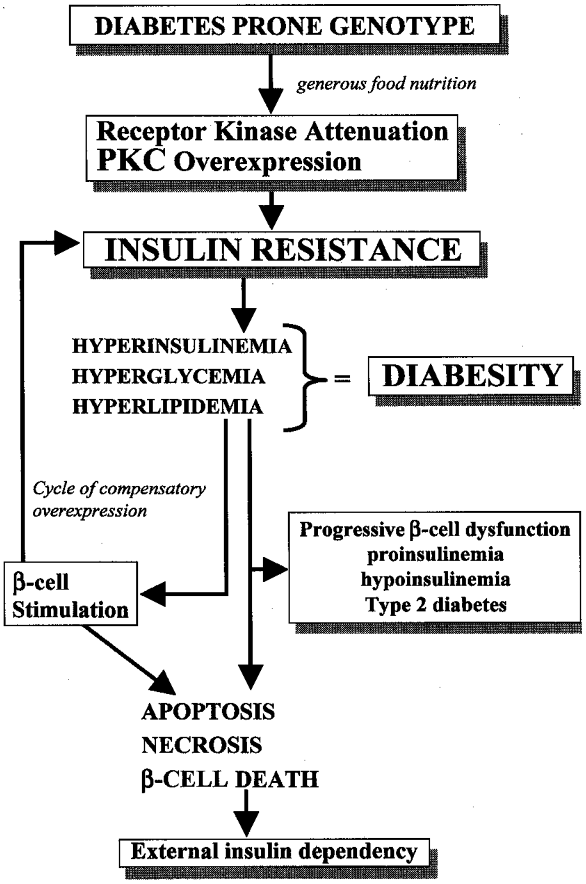

FIGURE 28 Schematic presentation of the development of nutritional diabetes in *Psammomys obesus* and subjects with a thrifty gene background consuming an abundant ad libitum diet. Insulin resistance and PKC$\varepsilon$ overexpression develop. Initially marginal hyperglycemia promotes $\beta$-cell insulin secretion resulting in hyperinsulinemia, which in time becomes insufficient to compensate for the rising glucose level. The hyperinsulinemia and PKC overexpression attenuate the function of insulin receptor and its substrate as well as the glucose transport and PKB activity. Tissue triglyceride deposition ensues with raised levels of DAG which in turn exacerbate the PKC$\varepsilon$ expression. The permanent secretion pressure on $\beta$-cells causes apoptosis and necrosis requiring support with external insulin for survival.

of the insulin resistance syndrome in animals and humans alike and may be therefore considered as "PKC overexpression syndrome".

Since the times of ancient discoveries of the causes of diabetes, emphasis was always placed on sweetness of urine, blood and other body fluids, leading to the addition of the adjective "mellitus" to diabetes. We should reconsider if this adjective is fully justified. The traditional concepts related to "sweetness" and emphasis on glucose-insulin axis do not explain the basic pathophysiological mechanisms leading to severe complications and mortality both in type 1 and type 2 diabetes as well as the reasons for the development of insulin resistance. The major causes of IGT and diabetes morbidity are strongly related to aberrant fat rather than carbohydrate metabolism. These include lack of restraint of the mobilization of FFA from adipose tissue leading to lipolysis and subsequent excessive fatty acid oxidation, acidosis and ketosis causing defect in glucose uptake by peripheral tissues as well as enhanced hepatic gluconeogenesis and delivery of fat to muscle. It may be remarked that the restraint of lipolysis in adipose tissue is the most sensitive action of insulin which becomes compromised by hyperinsulinemia. In blood vessels the oxidative trend prevailing in diabetes directs the cholesterol esterified with unsaturated fatty acids to surrogate receptors due to nonrecognition of the oxidized molecules, which facilitates foam cell and smooth muscle proliferation, arterial coronary cholesterol plaques, atherosclerosis and thrombosis. In the pancreas the excess of lipids taken up or synthesized in $\beta$-cells leads to $\beta$-cell malfunction due to lipotoxicity with apoptosis and necrosis. As indicated here and elsewhere the accumulation of muscle TG and the consequent rise in the content of the triglyceride intermediate DAG is instrumental in overexpression of PKC$\varepsilon$ and other PKC isoenzymes. In addition to the derangement in fat metabolism discussed in detail by McGarry[105] the newly discovered detrimental role of DAG accumulation may have uncovered a new culprit implicating fat metabolism as a causative metabolic deviation in diabetes. Perhaps this is the time to start using the term "diabetes lipidicus" rather than "mellitus" in order to appropriately define the diabetes pathophysiology.

### *Acknowledgement*

I would like to offer my sincere thanks to my colleagues, Drs. Ehud Ziv, Luitgard Mosthaf-Seedorf and Yukio Ikeda for their extensive contribution in the research of molecular biology of diabetes in *Psammomys obesus*.

### *References*

[1] Renold, A. E., Porte, D. Jr. and Shafrir, E. (1988). Definitions for diabetes types: use and abuse of the concept 'animal models of diabetes mellitus'. In: Shafrir, E. and Renold, A. E. (Eds.) *Lessons from Animal Diabetes*, J. Libbey & Company, London, **2**, 3–7.

[2] Rabinovitz, A., Gutzeit, A., Grill, V., Kikuchi, M., Renold, A. E. and Cerasi, E. (1975). Defective insulin secretion in the spiny mouse (*Acomys cahirinus*). Possible value in the study of the pathophysiology of diabetes. *Isr. J. Med. Sci.*, **11**, 730–737.

[3] Shafrir, E. (2000). Overnutrition in spiny mice (*Acomys cahirinus*): $\beta$-cell expansion leading to rupture and overt diabetes on fat-rich diet and protective energy-wasting elevation in thyroid hormone on sucrose-rich diet. *Diabetes Metab. Res. Rev.*, **16**, 94–105.

[4] Gutzeit, A., Renold, A. E., Cerasi, E. and Shafrir, E. (1979). Effect of diet-induced obesity on glucose and insulin tolerance of a rodent with a low insulin response (*Acomys cahirinus*). *Diabetes*, **28**, 777–784.

[5] Shafrir, E. (1982). Intermediary metabolism during the development of obesity and diabetes in the desert rodent *Acomys cahirinus*. *Int. J. Obes.*, **6**, 9–20.

[6] Polonsky, K., Sturis, J. and Bell, G. J. (1996). Non-insulin-dependent diabetes mellitus – A genetically programmed failure of the beta cell to compensate for insulin resistance. *N. Engl. J. Med.*, **334**, 777–783.

[7] Leiter, E. H. (1989). The genetics of diabetes susceptibility in mice. *FASEB J.*, **3**, 2231–2241.

[8] Leiter, E. H. (1987). Analysis of differential survival of syngeneic islets transplanted into hyperglycemic C57BL/6J *versus* C57BL/KsJ mice. *Transplantation*, **44**, 401–406.

[9] Hackel, D. B., Frohman, L. A., Mikat, E., Lebovitz, H. E., Schmidt-Nielsen, K. and Kinney, T. D. (1965). Review of the current studies on effect of diet on the glucose tolerance of the sand rat (*Psammomys obesus*). *Ann. NY. Acad. Sci.*, **131**, 459–463.

[10] Adler, J. H., Kalman, R., Lazarovici, G., Bar-On, H. and Ziv, E. (1991). Achieving predictable model of Type 2 diabetes in sand rats. In: Shafrir, E. (Ed.) *Lessons from Animal Diabetes*, Smith-Gordon, London, **3**, 212–214.

[11] Kalderon, B., Gutman, A., Shafrir, E. and Adler, J. H. (1986). Characterization of stages in the development of obesity-diabetes syndrome in sand rat. (*Psammomys obesus*) *Diabetes*, **35**, 717–724.

[12] Shafrir, E. and Gutman, A. (1993). *Psammomys obesus* of the Jerusalem colony: A model for nutritionally induced, non-insulin-dependent diabetes. *J. Basic Clin. Physiol. Pharmacol.*, **4**, 83–99.

[13] Ziv, E. and Shafrir, E. (1995). *Psammomys obesus*: nutritionally induced NIDDM-like syndrome on a 'thrifty gene' background. In: Shafrir, E. (Ed.) *Lessons from Animal Diabetes*, Smith-Gordon, London, **5**, 285–300.

[14] Shafrir, E. and Ziv, E. (1998). Cellular mechanism of nutritionally induced insulin resistance: The desert rodent *Psammomys obesus* and other animals in which insulin resistance leads to detrimental outcome. *J. Basic. Clin. Physiol. Pharmacol.*, **9**, 347–385.

[15] Marquie, G., Duhault, J. and Jacotot, B. (1984). Diabetes mellitus in sand rats (*Psammomys obesus*). Metabolic pattern during development of the diabetic syndrome. *Diabetes*, **33**, 438–443.

[16] Barnett, M., Collier, G. R., Collier, F. McL., Zimmet, P. and O'Dea, K. (1994). A cross-sectional and short-term longitudinal characterization of NIDDM in *Psammomys obesus*. *Diabetologia*, **37**, 671–676.

[17] Barnett, M., Collier, G. R., Zimmet, P. and O'Dea, K. (1995). Energy intake with respect to the development of diabetes mellitus in *Psammomys obesus*. *Diab. Nutr. Metab.*, **8**, 1–6.

[18] Park, J., Lemieux, S., Lewis, G. F., Kuksis, A. and Steiner, G. (1997). Chronic exogenous insulin and chronic carbohydrate supplementation increase de novo VLDL triglyceride fatty acid production in rats. *J. Lipid Res.*, **38**, 2529–2536.

[19] Collier, G. R., McMillan, J. S., Windmill, K., Walder, K., Tenne-Brown, J., deSilva, A., Trevakis, J., Jones, S., Morton, G. S., Lee, S., Augert, G., Civtarese, A. and Zimmet, P. Z. (2000). Beacon: a novel gene involved in regulation of energy balance. *Diabetes*, **49**, 1766–1771.

[20] Walder, K., McMillan, J. S., Lee, S., Civitarese, A., Zimmet, P. and Collier, G. R. (2001). Effects of beacon administration on energy expenditure and substrate utilization in *Psammomys obesus* (Israeli Sand rat). *Int. J. Obes.*, **25**, 1281–1285.

[21] Bar-On, H., Ben-Sasson, R., Ziv, E., Arar, N. and Shafrir, E. (1999). Irreversibility of nutritionally induced NIDDM in *Psammomys obesus* is related to $\beta$-cell apoptosis. *Pancreas*, **18**, 259–265.

[22] Barnett, M., Collier, G. R., Zimmet, P. and O'Dea, K. (1994). The effect of restricting energy intake on diabetes in *Psammomys obesus*. *Int. J. Obesity*, **18**, 789–794.

[23] Like, A. A. and Miki, E. (1967). Diabetic syndrome in sand rats. IV. Morphologic changes in islet tissue. *Diabetologia*, **3**, 143–146.

[24] Bendayan, M., Malide, D., Ziv, E., Levy, E., Ben-Sasson, R., Kalman, R., Chretien, M. and Seidah, N. G. (1993). Immunocyto-chemical investigation of insulin secretion by pancreatic β-cells in normal and diabetic *Psammomys obesus*. *J. Histochem. Cytochem.*, **43**, 771–784.

[25] Shafrir, E., Ben-Sasson, R., Ziv, E. and Bar-On, H. (1999). Insulin resistance, β-cell survival, and apoptosis in type 2 diabetes: animal models and human implications. *Diabetes Reviews*, **7**, 114–123.

[26] Donath, M. Y., Gross, D., Cerasi, E. and Kaiser, N. (1999). Hyperglycemia-induced β-cell apoptosis in pancreatic islets of *Psammomys obesus* during development of diabetes. *Diabetes*, **48**, 738–744.

[27] Nesher, R., Gross, D., Donath, M. Y., Cerasi, E. and Kaiser, N. (1999). Interaction between genetic and dietary factors determines β-cell function in *Psammomys obesus*, an animal model of type 2 diabetes. *Diabetes*, **48**, 731–737.

[28] Jörns, A., Ziv, E., Shafrir, E., Tiedge, M. and Lenzen, S. (2001). Gradual loss of pancreatic beta-cell insulin, glucokinase and GLUT2 glucose transporter immunoreactivities during the time course of nutritionally-induced type 2 diabetes in *Psammomys obesus* (sand rat). *Virchows Arch. A3Q* (in press).

[29] Pick, A., Clark, J., Kubstrup, C., Levisetti, M., Pugh, W., Bonner-Weir, S. and Polonsky, K. S. (1998). Role of apoptosis in failure of β-cell mass compensation for insulin resistance and β-cell defects in the male Zucker diabetic fatty rat. *Diabetes*, **47**, 358–364.

[30] Purrello, F., Vetri, M., Gatta, C., Gullo, D. and Vigneri, R. (1989). Effects of high glucose on insulin secretion by isolated rat islets and purified β-cells and possible role of glycosylation. *Diabetes*, **38**, 1417–1422.

[31] Eizirik, D. L., Korbutt, G. S. and Hellerstrom, C. (1992). Prolonged exposure of human pancreatic islets to high glucose concentrations *in vitro* impairs the β-cell function. *J. Clin. Invest.*, **90**, 1263–1268.

[32] Unger, R. H. (1995). Lipotoxicity in the pathogenesis of obesity-dependent NIDDM: Genetic and clinical implications. *Diabetes*, **44**, 863–870.

[33] Kaneto, H., Fujii, J., Myint, T. M., Miyazawa, N., Islam, K. N., Kawasaki, Y., Suzuki, K., Nakamura, M., Tatsumi, H., Yamasaki, Y. and Taniguchi, N. (1996). Reducing sugars trigger oxidative modification and apoptosis in pancreatic beta-cells by provoking oxidative stress through the glycation reaction. *Biochem. J.*, **320**, 855–863.

[34] Gadot, M. G., Leibowitz, G., Shafrir, E., Cerasi, E., Gross, D. J. and Kaiser, N. (1994). Hyperproinsulinemia and insulin deficiency in the diabetic *Psammomys obesus*. *Endocrinology*, **135**, 610–616.

[35] Ward, W. K., Lacava, E. C., Paquette, T. L., Beard, J. C., Wallum, B. J. and Porte, D. (1987). Disproportionate elevation of immunoreactive proinsulin in type 2 (non-insulin) diabetes mellitus and in experimental insulin resistance. *Diabetologia*, **30**, 698–702.

[36] Glauber, H. S., Revers, R. R., Henry, R., Schmeiser, L., Wallace, P., Kolterman, O. G., Cohen, R. M., Rubenstein, A. H., Galloway, J. A., Frank, B. H. and Olefsky, J. M. (1986). *In vivo* deactivation of proinsulin action on glucose disposal and hepatic glucose production in normal man. *Diabetes*, **35**, 311–317.

[37] Kanety, H., Moshe, S., Shafrir, E., Lunenfeld, B. and Karasik, A. (1994). Hyperinsulinemia induces a reversible impairment in insulin receptor function leading to diabetes in the sand rat model of non-insulin-dependent diabetes mellitus. *Proc. Natl. Acad. Sci. USA*, **91**, 1853–57.

[38] Marban, S. L., De Loia, J. A. and Gearhart, J. D. (1989). Hyperinsulinemia in transgenic mice carrying multiple copies of the human insulin gene. *Devel. Genetics*, **19**, 356–364.

[39] Tang, J., Neldigh, J. L., Cooksey, R. C. and McClain, D. A. (2000). Transgenic mice with increased hexosamine flux specifically targeted to β-cells exhibit hyperinsulinemia and peripheral insulin resistance. *Diabetes*, **49**, 1492–1499.

[40] Sone, H., Suzuki, H., Takahashi, A. and Yamada, N. (2001). Disease model: hyperinsulinemia and insulin resistance. Part A – targeted disruption of insulin signaling or glucose transport. Part B. *Trends Mol. Med.*, **7**, 320–322.

[41] Miles, P. D. G., Li, S., Hart, M., Romeo, O., Cheng, J., Cohen, A., Raafat, K., Moossa, A. R. and Olefsky, J. M. (1998). Mechanisms of insulin resistance in experimental hyperinsulinemic dogs. *J. Clin. Invest.*, **101**, 202–211.

[42] Treadway, H., Whitaker, J. and Pessin, J. E. (1989). Regulation of the insulin receptor kinase by hyperinsulinemia. *J. Biol. Chem.*, **266**, 15136–15143.

[43] Arsenis, G. and Livingston, J. M., Alterations in tyrosine kinase activity of the insulin receptor produced by *in vitro* hyperinsulinemia. *J. Biol. Chem.*, **261**, 147–153.

[44] Williams, J. F. and Olefsky, J. M. (1990). Defective insulin receptor function in down-regulated HepG2 cells. *Endocrinology*, **127**, 1706–1717.

[45] McNulty, P. H., Louard, R., Deckelbaum, R. J., Zaret, L. I. and Young, L. H. (1995). Hyperinsulinemia inhibits myocardial protein degradation in patients with cardiovascular disease and insulin resistance. *Circulation*, **92**, 2151–2156.

[46] Del Prato, S., Leonetti, F., Simonson, D. C., Sheehan, P., Matsuda, M. and DeFronzo, R. A. (1994). Effect of sustained physiologic hyperinsulinaemia and hyperglycaemia on insulin secretion and insulin sensitivity in man. *Diabetologia*, **27**, 1025–1035.

[47] Pontiroli, A. E., Alberetto, M. and Pozza, G. (1992). Patients with insulinoma show insulin resistance in the absence of arterial hypertension. *Diabetologia*, **35**, 294–295.

[48] Weyer, C., Hanson, R. L., Tataranni, P. A., Bogardus, C. and Pratley, R. E. (2000). A high fasting plasma insulin concentration predicts type 2 diabetes independent of insulin resistance. *Diabetes*, **49**, 2094–2101.

[49] Ikeda, Y., Olsen, G. S., Ziv, E., Hansen, L. L., Busch, A. K., Hansen, B. F., Shafrir, E. and Mosthaf-Seedorf, L. (2001). Cellular mechanism of nutritionally induced insulin resistance in *Psammomys obesus*. Overexpression of protein kinase Cε in skeletal muscle precedes the onset of hyperinsulinemia and hyperglycemia. *Diabetes*, **50**, 584–592.

[50] Shafrir, E., Ziv, E. and Mosthaf, L. (1999). Nutritionally induced insulin resistance and receptor defect leading to β-cell failure in animal models. *Annals NY. Acad. Sci.*, **892**, 223–246.

[51] Haring, H. U., Kellerer, M. and Mosthaf, L. (1994). Modulation of insulin signaling in non-insulin-dependent diabetes mellitus: significance of altered receptor isoform patterns and mechanisms of glucose-induced receptor modulation. *Horm. Res.*, **41**(Suppl. 2), 87–92.
[52] Nishizuka, Y. (1995). Protein kinase C and lipid signaling for sustained cellular responses. *FASEB J.*, **9**, 484–496.
[53] Kalman, R., Adler, J. H., Lazarovici, G., Bar-On, H. and Ziv, E. (1993). The efficiency of sand rat metabolism is responsible for development of obesity and diabetes. *J. Basic Clin. Physiol. Pharmacol.*, **4**, 83–99.
[54] Ziv, E., Kalman, R., Hershkop, K., Barash, V., Shafrir, E. and Bar-On, H. (1996). Insulin resistance in the NIDDM model *Psammomys obesus* in the normoglycemic, normoinsulinemic state. *Diabetologia*, **39**, 1269–1275.
[55] Berti, L., Mosthaf, L., Kroder, G., Kellerer, M., Tippmer, S., Mushak, J., Seffer, E., Seedorf, K. and Haring, H. (1994). Glucose-induced translocation of protein kinase C isoforms in rat-1 fibroblasts is paralleled by inhibition of the insulin receptor tyrosine kinase. *J. Biol. Chem.*, **369**, 3381–3386.
[56] Danielsen, A. G., Liu, F., Hosomi, Y., Shii, K. and Roth, R. A. (1995). Activation of protein kinase C$\alpha$ inhibits signaling by members of the insulin receptor family. *J. Biol. Chem.*, **270**, 21600–21605.
[57] Bossenmaier, B., Mosthaf, L., Mischak, H., Ullrich, A. and Haring, H. U. (1997). Protein kinase C isoforms $\beta 1$ and $\beta 2$ inhibit the tyrosine kinase activity of the insulin receptor. *Diabetologia*, **40**, 863–866.
[58] Tanti, J. F., Gremeaux, T., van Obberghen, E. and Le Marchand Brustel, Y. (1994). Serine/threonine phosphorylation of insulin receptor substrate 1 modulates insulin receptor signaling. *J. Biol. Chem.*, **269**, 6051–6057.
[59] Karasik, A., Rothenberg, P. L., Yamada, K., White, M. F. and Kahn, C. R. (1990). Increased protein kinase C activity is linked to reduced insulin receptor autophosphorylation in liver of starved rats. *J. Biol. Chem.*, **265**, 10226–10231.
[60] Kellerer, M., Mushack, J., Seffer, E., Mischak, H., Ullrich, A. and Haring, H. U. (1998). Protein kinase C isoforms $\alpha$, $\delta$, and $\theta$ require insulin receptor substrate-1 to inhibit the tyrosine kinase activity of the insulin receptor in human kidney embryonic cells (HEK 293 cells). *Diabetologia*, **41**, 833–838.
[61] Seedorf, K., Shearman, M. and Ullrich, A. (1995). Rapid and long term effects of protein kinase C on receptor tyrosine kinase phosphorylation and degradation. *J. Biol. Chem.*, **270**, 18953–18960.
[62] Chin, J. E., Liu, F. and Roth, R. A. (1994). Activation of protein kinase C$\alpha$ inhibits insulin-stimulated tyrosine phosphorylation of insulin receptor substrate-1. *Molecular Endocrinology*, **8**, 51–58.
[63] Dunaif, A., Xia, J. R., Book, C. B., Schenker, E. and Tang, Z. C. (1995). Excessive insulin receptor serine phosphorylation in cultured fibroblasts and in skeletal muscle – a potential mechanism for insulin resistance in the polycystic ovary syndrome. *J. Clin. Invest.*, **96**, 801–810.
[64] deVente, J. E., Carey, J. O., Bryant, W. O., Pettit, G. J. and Ways, D. K. (1996). Transcriptional regulation of insulin receptor substrate 1 by protein kinase C. *J. Biol. Chem.*, **271**, 32276–32280.
[65] Kellerer, M., Mushack, J., Mischak, H. and Häring, H. U. (1997). Protein kinase C (PKC) epsilon enhances the inhibitory effect of TNF alpha on insulin signaling in HEK293 cells. *FEBS Lett.*, **418**, 119–122.
[66] Hotamisligil, G. S., Murray, D., Choy, L. N. and Spiegelman, B. M. (1994). Tumor necrosis factor $\alpha$ inhibits signaling from the insulin receptor. *Proc. Natl. Acad. Sci. USA*, **91**, 4854–4858.
[67] Rui, L. Y., Aguirre, V., Kim, J. K., Shulman, G. I., Lee, A., Corbould, A., Dunaif, A. and White, M. F. (2001). Insulin/IGF-1 and TNF-alpha stimulate phosphorylation of IRS-1 at inhibitory Ser (307) *via* distinct pathways. *J. Clin. Invest.*, **107**, 181–189.
[68] Strack, V., Hennige, A. M., Krutzfeldt, J., Bossenmaier, B., Klein, H.-H., Kellerer, M., Lammers, R. and Häring, H. U. (2000). Serine residues 994 and 1023/25 are important for insulin receptor kinase inhibition by protein kinase C isoforms $\beta 2$ and $\theta$. *Diabetologia*, **43**, 443–449.
[69] De Fea, K. and Roth, R. A. (1997). Protein kinase C modulation of insulin receptor substrate-1 tyrosine phosphorylation requires serine 612. *Biochemistry*, **36**, 12939–12947.
[70] Al-Hasani, H., Eisermann, B., Tennagels, N., Magg, C., PaBlack, W., Koenen, M., Muller-Wieland, D., Meyer, H. E. and Klein, H. W. (1997). Identification of Ser-1275 and Ser-1309 as autophosphorylation sites of the insulin receptor. *FEBS Letters*, **400**, 65–70.
[71] Avignon, A., Yamada, K., Zhou, X., Spencer, B., Cardona, O., Saba-Siddique, S., Galloway, L., Standaert, M. L. and Farese, R. V. (1996). Chronic activation of protein kinase C in soleus muscle and other tissues of insulin-resistant type II diabetic Goto-Kakizaki (GK) obese/aged, and obese/Zucker rats: a mechanism for inhibiting glycogen synthesis. *Diabetes*, **45**, 1396–1404.
[72] Chen, K. S., Heydrick, S. J., Brown, M. L., Friel, J. C. and Ruderman, N. B. (1994). Insulin increases a biochemically distinct pool of diacylglycerol in the rat soleus muscle. *Am. J. Physiol.*, **266**, E479–E485.
[73] Yu, H. Y., Inoguchi, T., Kakimoto, M., Nakashima, N., Imammura, M., Hashimoto, T., Umeda, F. and Nawata, H. (2001). Saturated non-esterified fatty acids stimulate de novo diacylglycerol synthesis and protein kinase c activity in cultured aortic smooth muscle cells. *Diabetologia*, **44**, 614–20.
[74] Li, M. and Bjorntorp, P. (1995). Triglyceride uptake in muscles in rats. *Obesity Res.*, **3**, 419–426.
[75] Griffin, M. E., Marcucci, M. J., Cline, G. W., Bell, K., Barucci, N., Lee, D., Goodyear, L. J., Kraegen, E. W., White, M. F. and Shulman, G. I. (1999). Free fatty acid-induced insulin resistance is associated with activation of protein kinase C$\theta$ and alterations in the insulin signaling cascade. *Diabetes*, **48**, 1270–1274.
[76] Ebeling, P., Essen-Gustavsson, B., Tuominen, J. A. and Koivisto, V. A. (1998). Intramuscular triglyceride content is increased in IDDM. *Diabetologia*, **41**, 111–115.
[77] Kraegen, E. W., Clark, P. W., Jenkins, A. B., Daley, E., Chisholm, D. J. and Storlien, L. H. (1991). Muscle insulin resistance develops after liver insulin resistance in the high fat fed rat. *Diabetes*, **40**, 1397–1403.
[78] Kraegen, E. W., Carey, D. G. P. and Campbell, L. V. (2000). Effects of lipids on blood glucose regulation and insulin action. In: Draznin, B. and Rizza, R. (Eds.) *Clinical Research in Diabetes and Obesity, Part I. Methods, Assessment and Metabolic Regulation.* (pp. 305–320), Humana Press Inc., NJ.
[79] Schmitz-Peiffer, C., Browne, C. L., Oakes, N. D., Watkinson, A., Chisholm, D. J., Kraegen, E. W. and Biden, T. J. (1997). Alterations in the expression and cellular localization of protein kinase C isozymes $\varepsilon$ and $\theta$ are associated with insulin resistance in skeletal muscle of the high-fat-fed rat. *Diabetes*, **46**, 169–178.

[80] Pan, D. A., Lillioja, S., Kriketos, A. D., Milner, M. R., Baur, L. A., Bogardus, C., Jenkins, A. B. and Storlien, L. H. (1997). Skeletal muscle triglyceride levels are inversely related to insulin action. *Diabetes*, **46**, 983–988.

[81] Goldstein, B. J. (2000). Protein-tyrosine phosphatases and the regulation of insulin action, In: LeRoith, D. J., Olefsky, M. and Taylor, S. I. (Eds.) *Diabetes Mellitus. A Fundamental and Clinical Text*. 2nd Edition (pp. 206–217). Lippincott, Philadelphia.

[82] Goldstein, B. J., Ahmad, F., Ding, W., Li, P.-M. and Zhang, W.-R. (1998). Regulation of the insulin signaling pathway by cellular protein-tyrosine phosphatases. *Mol. Cell Biochem.*, **182**, 91–99.

[83] Goldstein, B. J., Bittner-Kowalczyk, A., White, M. F. and Harbeck, M. (2000). Tyrosine dephosphorylation and deactivation of insulin receptor substrate-1 by protein phosphatase 1B. Possible facilitation by the formation of a ternary complex with the GRB2 adaptor protein. *J. Biol. Chem.*, **275**, 4283–4289.

[84] Seely, B. S., Staubs, P. A., Reichart, D. R., Berhanu, P., Milarski, K. L., Saltiel, A. R., Kusari, J. and Olefsky, J. M. (1996). Protein tyrosine phosphatase 1B interacts with the activated insulin receptor. *Diabetes*, **45**, 1379–1385.

[85] Kenner, K. A., Anyanwu, E., Olefsky, J. M. and Kusari, J. (1996). Protein-tyrosine phosphatasse 1B is a negative regulator of insulin- and insulin-like growth factor-I-stimulated signaling. *J. Biol. Chem.*, **271**, 19810–19816.

[86] Olichon-Berthe, C., Hauguel-De Mouzon, S., Peraldi, P., van Obberghen, E. and Le Marchand-Brustel, Y. (1994). Insulin receptor dephosphorylation by phosphotyrosine phosphatases obtained from insulin-resistant obese mice. *Diabetologia*, **37**, 56–60.

[87] Meyerovitch, J., Backen, J. M. and Kahn, C. R. (1989). Hepatic phosphotyrosine phosphatase activity and its alteration in diabetic rats. *J. Clin. Invest.*, **84**, 976–983.

[88] Sredy, J., Savicki, D. R., Flam, B. R. and Sullivan, D. (1995). Insulin resistance is associated with abnormal dephosphorylation of synthetic phospopeptide corresponding to the major autophosphorylation sites of the insulin receptor. *Metabolism*, **44**, 1074–1081.

[89] Ahmad, F. and Goldstein, B. J. (1995). Increased abundance of specific skeletal muscle protein-tyrosine phosphatases in a genetic model of insulin-resistant obesity and diabetes mellitus. *Metabolism*, **44**, 1175–1184.

[90] Meyerovitch, J., Balta, Y., Goldstein, B. J., Ziv, E., Kalman, R., Sack, J. and Shafrir, E., *Psammomys obesus* (a rodent model of insulin resistance) has an altered regulation and activity of protein-tyrosine phosphatase. Submitted for publication.

[91] Prochazka, M., Mochizuki, H., Baier, L. J., Cohen, P. T. W. and Bogardus, C. (1995). Molecular and linkage analysis of type-1 protein phosphatase catalytic beta-subunit gene: lack of evidence for its major role in insulin resistance in Pima Indians. *Diabetologia*, **38**, 461–466.

[92] Ikeda, Y., Ziv, E., Shafrir, E. and Mosthaf-Seedorf, L. (2001). Protein tyrosine phosphatase 1B is impaired in skeletal muscle of diabetic *Psammomys obesus*. *Diabetes Res. Clin. Pract.*, **50**(Suppl. 1), S156–157.

[93] Worm, D., Handberg, A., Hoppe, E., Vinten, J. and Beck-Nielsen, H. (1996). Decreased skeletal muscle phosphotyrosine phosphatase (PTPase) activity towards insulin receptors in insulin-resistant Zucker rats measured by delayed Europium fluorescence. *Diabetologia*, **39**, 142–148.

[94] Bursell, S. E., Takagi, C., Clermont, A. C., Takagi, H., Mori, F., Ishii, H. and King, G. L. (1997). Specific retinal diacylglycerol and protein kinase C beta isoform modulation mimics abnormal retinal hemodynamics in diabetic rats. *Invest. Ophthal. Vis. Sci.*, **38**, 2711–2720.

[95] Bursell, S.-E. and King, G. L. (2000). Protein kinase C activation, development of diabetic vascular complications, and role of vitamin E in preventing these abnormalities. In: Packer, L. *et al.* (Eds.) *Antioxidants in Diabetes Management* (pp. 241–263). Marcel Dekker, NY.

[96] Muller, H. K., Kellerer, M., Ermel, B., Muhlhofer, B., Obermaier-Kusser, B., Vogt, B. and Häring, H. U. (1991). Prevention by protein kinase C inhibitors of glucose-induced insulin-receptor tyrosine kinase resistance in rat fat cells. *Diabetes*, **40**, 1440–1448.

[97] Taher, M. M. (1996). Herbimycin A inhibits protein kinase C in vascular smooth muscle cells. *Biochem. Molec. Biol. Int.*, **39**, 267–277.

[98] Koya, D. and King, G. L. (1998). Protein kinase C activation and the development of diabetic complications. *Diabetes*, **47**, 859–866.

[99] Kim, J. K., Fillmore, J., Sunshine, M. J., Chen, Y., Zong, H., Littman, D. R. and Shulman, G. I. (2001). Transgenic mice with inactivation of PKC-$\theta$ are protected from lipid-induced defects in insulin action and signaling in skeletal muscle. *Diabetes*, **50**(Suppl. 1), A58.

[100] Qureshi, S. A., Ding, V., Li, Z. H., Szalkowski, D., Biazzo-Ashnault, D. E., Xie, D., Saperstein, R., Brady, E., Huskey, S., Shen, X. L., Liu, K., Xu, L. B., Salituro, G. M., Heck, J. V., Moller, D. E., Jones, A. B. and Zhang, B. B. (2000). Activation of insulin signal transduction pathway and anti-diabetic activity of small molecule insulin receptor activators. *J. Biol. Chem.*, **275**, 3543–3549.

[101] Shafrir, E., Spielman, S., Nachliel, I., Khamaisi, M., Bar-On, H. and Ziv, E. (2001). Treatment of diabetes with vanadium salts: general overview and amelioration of nutritionally induced diabetes in the *Psammomys obesus* gerbil. *Diabetes Metab. Res. Rev.*, **17**, 55–66.

[102] Cam, M. D., Brownsey, R. W. and McNeill, J. H. (2000). Mechanisms of vanadium action: insulin-mimetic or insulin-enhancing agent? *Can. J. Physiol. Pharmacol.*, **78**, 829–847.

[103] Goldwaser, I., Li, J., Gershonov, E., Armoni, M., Karnieli, E., Fridkin, M. and Shechter, Y. (1999). L-Glutamic acid $\gamma$-monohydroxamate. A potentiator of vanadium evoked glucose metabolism *in vitro* and *in vivo*. *J. Biol. Chem.*, **27**, 26617–26624.

[104] Brichard, S. M. and Henquin, J. C. (1995). The role of vanadium in the management of diabetes. *Trends Pharmacol. Sci.*, **16**, 265–279.

[105] McGarry, J. D. (1992). What if Minkowski had been ageusic? An alternative angle on diabetes. *Science*, **258**, 766–770.

